# Oxysterole-binding protein targeted by SARS-CoV-2 viral proteins regulates coronavirus replication

**DOI:** 10.3389/fcimb.2024.1383917

**Published:** 2024-07-25

**Authors:** Yue Ma-Lauer, Pengyuan Li, Daniela Niemeyer, Anja Richter, Konstantin Pusl, Brigitte von Brunn, Yi Ru, Chengyu Xiang, Sebastian Schwinghammer, Jia Liu, Priya Baral, Emilia J. Berthold, Haibo Qiu, Avishek Roy, Elisabeth Kremmer, Heinrich Flaswinkel, Christian Drosten, Zhendong Jin, Albrecht von Brunn

**Affiliations:** ^1^ Virology Department, Max-von-Pettenkofer Institute, Ludwig-Maximilians-University Munich, Munich, Germany; ^2^ German Center for Infection Research (DZIF), Munich Site, Munich, Germany; ^3^ Institute of Virology, Campus Charité Mitte, Charité - Universitätsmedizin Berlin, Berlin, Germany; ^4^ German Centre for Infection Research, Associated Partner Charité, Berlin, Germany; ^5^ Institute of Lung Health and Immunity and Comprehensive Pneumology Center with the Comprehensive Pneumology Center Munich (CPC-M) bioArchive, Helmholtz-Zentrum München, Munich, Germany; ^6^ Department of Pharmaceutical Sciences and Experimental Therapeutics, College of Pharmacy, The University of Iowa, Iowa City, IA, United States; ^7^ BioSysM, Ludwig-Maximilians-University Munich, Munich, Germany

**Keywords:** OSBP, SARS-CoV-2, coronavirus, Nsp3, Nsp4, Nsp6, VAP-B

## Abstract

**Introduction:**

Oxysterol-binding protein (OSBP) is known for its crucial role in lipid transport, facilitating cholesterol exchange between the Golgi apparatus and endoplasmic reticulum membranes. Despite its established function in cellular processes, its involvement in coronavirus replication remains unclear.

**Methods:**

In this study, we investigated the role of OSBP in coronavirus replication and explored the potential of a novel OSBP-binding compound, ZJ-1, as an antiviral agent against coronaviruses, including SARS-CoV-2. We utilized a combination of biochemical and cellular assays to elucidate the interactions between OSBP and SARS-CoV-2 non-structural proteins (Nsps) and other viral proteins.

**Results:**

Our findings demonstrate that OSBP positively regulates coronavirus replication. Moreover, treatment with ZJ-1 resulted in reduced OSBP levels and exhibited potent antiviral effects against multiple coronaviruses. Through our investigation, we identified specific interactions between OSBP and SARS-CoV-2 Nsps, particularly Nsp3, Nsp4, and Nsp6, which are involved in double-membrane vesicle formation—a crucial step in viral replication. Additionally, we observed that Nsp3 a.a.1–1363, Nsp4, and Nsp6 target vesicle-associated membrane protein (VAMP)-associated protein B (VAP-B), which anchors OSBP to the ER membrane. Interestingly, the interaction between OSBP and VAP-B is disrupted by Nsp3 a.a.1–1363 and partially impaired by Nsp6. Furthermore, we identified SARS-CoV-2 orf7a, orf7b, and orf3a as additional OSBP targets, with OSBP contributing to their stabilization.

**Conclusion:**

Our study highlights the significance of OSBP in coronavirus replication and identifies it as a promising target for the development of antiviral therapies against SARS-CoV-2 and other coronaviruses. These findings underscore the potential of OSBP-targeted interventions in combating coronavirus infections.

## 1 Introduction

Coronaviruses, characterized by their single-stranded, plus-stranded RNA genomes, have the largest known viral RNA genomes, spanning over 27 kb (Gorbalenya et al., 2006). Historically, coronaviruses caused mild upper respiratory tract infections until the emergence of severe acute respiratory syndrome coronavirus (SARS-CoV) in 2002 and 2003, which demonstrated their potential to cause severe disease and initiate pandemics by jumping from animal to human populations (Drosten et al., 2003; Knipe and Howley, 2013). Following the SARS-CoV outbreak, another coronavirus, Middle East Respiratory Syndrome Coronavirus (MERS-CoV), emerged in Saudi Arabia in 2012, causing severe disease and highlighting the ongoing threat posed by coronaviruses (Zaki et al., 2012). Although the SARS-CoV pandemic waned after affecting over 8000 patients worldwide and resulting in 774 deaths (Chan-Yeung and Xu, 2003), MERS-CoV remains a significant public health concern, with 858 reported deaths as of September 2019 (WHO, 2019), mostly in the Middle East. However, the emergence of SARS-CoV-2, the causative agent of coronavirus disease 2019 (COVID-19), has created an unprecedented global health crisis, resulting in millions of deaths within three years (WHO, 2023). While the World Health Organization no longer considers COVID-19 to be a public health emergency of international concern as of May 2023, it remains an ongoing global health threat, exacerbated by the emergence of diverse SARS-CoV-2 variants and the potential for zoonotic transmission.

Oxysterol-binding protein (OSBP) serves as a critical lipid transporter mediating cholesterol/phosphatidylinositol-4-phosphate (PI4P) exchange between the Golgi apparatus and the endoplasmic reticulum (ER) in uninfected cells (Mesmin et al., 2013). In cells infected with certain single-stranded RNA viruses, such as enteroviruses, cardioviruses and hepatitis C virus (HCV), OSBP assumes an additional pivotal role by providing cholesterol to double-membrane vesicles (DMVs), essential replication organelles (ROs) for these viruses (Nagy et al., 2016). For example, HCV requires OSBP and phosphatidylinositol 4-kinase PI4KA, which generates PI4P, for replication. In HCV-infected Huh 7.5.1 cells, cholesterol trafficking to ROs (DMVs) was disturbed if OSBP or PI4KA was knocked down (Wang et al., 2014). Similar to HCV, encephalomyocarditis virus (EMCV) replication was suppressed by knocking down OSBP, which delivers cholesterol to EMCV ROs, or by knocking down PI4KA, which is recruited to ROs via binding to EMCV nonstructural protein 3A (Dorobantu et al., 2015). However, not all of the (+) ssRNA viruses are dependent on OSBP. Aphthovirus was insensitive to either OSBP inhibitor Itraconazole (ITZ) (Strating et al., 2015) or the PI4K inhibitor AL-9 (Dorobantu et al., 2015), suggesting the existence of an alternate mechanism to supply lipid to ROs. DMVs, whose morphogenesis requires OSBP-mediated cholesterol delivery, serve as ROs not only for HCV (Tabata et al., 2021), but also for coronaviruses (Limpens et al., 2011; Romero-Brey et al., 2012; Snijder et al., 2020). Given its involvement in cholesterol shuttling between the ER and DMVs, OSBP emerges as a potential target for broad-spectrum antiviral drugs.

Previous studies have demonstrated the anticancer and antiviral properties of OSBP-binding compounds against enteroviruses and HCV (Amako et al., 2009; Albulescu et al., 2015; Strating et al., 2015; Albulescu et al., 2017; Roberts et al., 2019). For example, OSW-1, a natural compound with high affinity for OSBP, effectively inhibited enteroviruses in various cell types (Albulescu et al., 2015). In addition, OSW-1 was found to be the only OSBP-binding compound that reduced OSPB protein levels in HEK293 and HCT116 cells and had prophylactic antiviral activity against enterovirus infection in HeLa cells, when it was compared with other OSBP-binding compounds ITZ, TTP-8307 and T-00127-HEV2 (THEV) (Roberts et al., 2019). ITZ exerted antiviral activity against not only coxsackievirus B3 (CVB3), enterovirus 71 (EV71) and EMCV in BGM cells but also human rhinovirus 14 and Saffold virus in HeLa R19 cells. ITZ binding to OSBP also inhibited sterol and PI4P transfer (Strating et al., 2015). TTP-8307 suppressed viral replication of CVB3 and EMCV in HeLa and HCV in Huh 9–13 cells (Albulescu et al., 2017). Interestingly, overexpression of OSBP in HeLa R19 cells could rescue the inhibitory effect of OSW-1 and ITZ but not TTP-8307 against CVB3 replication (Strating et al., 2015; Albulescu et al., 2017).

In this study, we investigate the role of OSBP in coronavirus infection, with a particular focus on SARS-CoV-2. We identify a novel OSBP-binding compound, ZJ-1, a structurally simplified analogue of the natural product OSW-1 (Zhou et al., 2005), and demonstrate its potent antiviral activity. In addition, we elucidate the multiple interactions between OSBP and various SARS-CoV-2 viral proteins, highlighting OSBP as a promising target for the development of antiviral drugs against coronaviruses.

## 2 Materials and methods

### 2.1 Plasmids and constructs

For the GFP-OSBP and HA-OSBP constructs, the OSBP gene was amplified by PCR using OSBP gateway primers (**Table S1**) from a pLJM1-FLAG-GFP-OSBP vector [gift from Roberto Zoncu (Addgene plasmid # 134659; http://n2t.net/addgene:134659; RRID: Addgene_134659)] (Lim et al., 2019) and cloned into a pDONR207 gateway vector via BP reaction to generate pDONR207-OSBP. OSBP was further cloned from pDONR207-OSBP into pDEST-GFP and pDEST-N-tag-HA gateway compatible destination vectors via LR reaction as previously described (Ma-Lauer et al., 2016) to generate pDEST-GFP-OSBP and pDEST-HA-OSBP gateway constructs, respectively. For OSBP knockout constructs, the oligo-dimers of OSBP gRNA1 or gRNA2 primers (**Table S1**) were cloned into a pLentiCrispr v2 vector (gift from Feng Zhang [Addgene plasmid # 52961; http://n2t.net/addgene:52961; RRID: Addgene_52961]) (Sanjana et al., 2014) via BsmBI sites.

For yeast 2-hybrid assays, SARS-CoV-2 genes were amplified from the SARS-CoV-2 genome cDNA using the appropriate primers (**Table S1**) (Thao et al., 2020) and cloned into pDONR207 vectors via BP reaction to generate constructs of pDONR207-SARS-CoV-2 genes. The transmembrane domain-deleted version of S2 (Δa.a.1206–1254) was generated based on pDONR207-SARS-CoV-2-S2 via Q5 Site-Directed Mutagenesis Kit (New England Biolabs, E0554S) according to the standard protocol provided by the manufacturer (primers used are listed in **Table S1**). The resulting pDONR207-SARS-CoV-2 genes and pDONR207-OSBP were cloned into yeast gateway destination vectors pGBKT7g and pGADT7g (Stellberger et al., 2012) via LR reaction.

For split YFP constructs, OSBP was cloned from pDONR207-OSBP into a pDEST-c-myc-YFP^N^ gateway compatible vector via LR reaction to generate YFP^N^-OSBP construct as previously described (Ma-Lauer et al., 2016). The VAP-B gene was cloned from pDONR223-VAP-B (Pfefferle et al., 2011) into pDEST-c-myc-YFP^N^ via LR reaction to generate the YFP^N^-VAP-B construct. SARS-CoV-2 Nsp4, Nsp6, orfM, orf3a, orf3b, orf7a, and orf7b genes were cloned from their corresponding pDONR207-SARS-CoV-2 plasmids into a pDEST-HA-YFP^C^ vector (Ma-Lauer et al., 2016) via LR reaction to generate HA-YFP^C^ viral gene fusion constructs.

For split-NanoBit assays, the VAP-B gene was PCR amplified using the appropriate primers (**Table S1**), and the VAP-B PCR product was inserted into pBiT1.1N LgBiT vector (Promega, #N2014) via EcoRI/XbaI cleavage sites to generate pLgBiT-VAP-B constructs. Since no proper cleavage sites provided by pBiT2.1N SmBiT vector (Promega, #2014) could be used to clone OSBP into pBiT2.1N SmBiT vector via restriction enzyme, OSBP was cloned in an alternative way. First, the rfB cassette from the pDEST-GFP Gateway destination vector was amplified using the appropriate primers (**Table S1**). The resulting rfB PCR fragment was then inserted into pBiT2.1N SmBiT via XhoI/XbaI cleavage sites to generate a pSmBiT gateway compatible destination vector. Finally, the OSBP gene was cloned from pDONR207-OSBP into the newly constructed pSmBiT Gateway destination vector via LR reaction to generate pSmBiT-OSBP.

For other plasmids used in this study, SARS-CoV-2 genes were cloned from the corresponding pDONR207-SARS-CoV-2 gene constructs into a pDEST-RFP gateway destination vector (Ma-Lauer et al., 2016) to generate pDEST-RFP-SARS-CoV-2 genes. The SARS-CoV-2 Nsp3 a.a.1–1363 expressing vector pCG-SARS-CoV-2 Nsp3 a.a.1–1363 was kindly provided by Konstantin Sparrer, Ulm, Germany. For the pSmBiT-GFP construct, GFP was amplified using the appropriate primers (**Table S1**) and subsequently cloned into the pDONR207 vector via BP reaction. An LR reaction was performed on the resulting pDONR207-GFP and pSmBiT Gateway vector to generate pSmBiT-GFP.

### 2.2 Cell culture and transfection

Caco-2 cells were cultured in Dulbecco’s modified Eagle’s medium (DMEM; Gibco) containing 20% (v/v) fetal bovine serum (FBS), 1% sodium pyruvate, 1% non-essential amino acids, and 1% penicillin/streptomycin. Huh7, BHK-21, L929, and HEK293 cells were cultured in DMEM containing 10% (v/v) FBS and 1% penicillin/streptomycin. All cell lines were grown in a humid atmosphere with 5% CO2 at 37°C.

For plasmid transfection into HEK293 or Huh7 cells, cells were seeded on appropriate culture plates to reach 70% to 80% confluence for transfection on the second day. Transfection was performed using Lipofectamine 3000 (Thermofisher) according to the manufacturer’s standard protocol.

### 2.3 Generation of OSBP knockout pools

The successfully cloned pLentiCrispr v2 OSBP gRNA1 or gRNA2 were co-transfected with psPAX2 (gift from Didier Trono [Addgene plasmid # 12260; http://n2t.net/addgene:12260; RRID: Addgene_12260]) and pCMV-VSV-G (gift from Bob Weinberg [Addgene plasmid # 8454; http://n2t.net/addgene:8454; RRID: Addgene_8454]) (Stewart et al., 2003), plasmids into HEK293T cells to produce Crispr OSBP gRNA lentiviruses. Three days post-transfection, the cell culture supernatant containing lentiviruses was harvested and applied to infect Huh7 cells in a 6-well plate. Forty-eight hours after infection, the Huh7 cells were selected with DMEM growth medium containing 10% (v/v) FBS, 1% Penicillin/Streptomycin and 2 µg/ml puromycin for 5 days. Surviving cell pools were used for Western blot as well as viral infection assays.

### 2.4 Viral infections and inhibition

Cells at approximately 70% confluence in culture plates were washed with phosphate-buffered saline (PBS) and then inoculated with virus-containing serum-free DMEM for 1.5 hours at 33°C with 5% CO_2_. The inoculum was then removed. Cells were washed with PBS and re-cultured in growth medium at 33°C with 5% CO_2_ for an additional 24, 48, or 72 hours.

A 40 mM ZJ-1 stock solution was prepared by dissolving 1 mg of the sodium salt of ZJ-1 in 28.44 µl dimethyl sulfoxide (DMSO). The stock solution was then further diluted in growth medium at various concentrations for viral inhibition assays.

For the plaque titration assay, SARS-CoV-2 (BetaCoV/Munich/ChVir984/2020 (B.1, EPI_ISL_406862) plaque reduction titers were determined by plaque titration experiments (n=2) from supernatants of Caco-2 cells treated with ZJ-1. 175,000 Vero E6 cells were seeded in a 24-well plate one day before infection. After washing with PBS, the cells were inoculated with 200 µl of serially diluted cell culture supernatants from infected cells at MOI 0.1. After 1 hour of adsorption at 37°C, the virus dilutions were removed and 500 μL of high-viscosity overlay (1:1 mixture of 2.4% Avicel and 2x concentrated DMEM supplemented with 5% FBS, 2% non-essential amino acids (#11140–035, Gibco), and 2% sodium pyruvate (#11360–039, Gibco) was added to each well. The overlay was discarded at 3 dpi and cells were fixed in 6% formaldehyde for 30 minutes, washed with PBS, and stained with crystal violet solution (Sigma Aldrich) for 15 minutes. Plaques were counted from one to two dilutions where clear plaques were detectable (Niemeyer et al., 2022).

### 2.5 Antibodies and drug

Mouse antibody 1H11 (1:400) recognizing HCoV-229E/-NL63 N-protein was purchased from INGENASA, Spain. Rabbit anti-OSBP (HPA039227, 1:1,000) and mouse anti-vinculin (V9264, 1:1,000) were purchased from Sigma-Aldrich. Rat anti-HA antibody (1:1,000) was purchased from Roche (11867423001). Rabbit polyclonal anti-GFP (1:1.000) was purchased from Invitrogen (A6455). Mouse anti-RFP (1:1.000) was purchased from Invitrogen (MA5–15257). Mouse anti-PDI (1:100) was purchased from Invitrogen (MA3–019). Mouse anti-dsRNA (1:1,000) was purchased from SCICONS, Hungary (J2), and anti-β-actin antibody (1:100,000) was provided by Sigma-Aldrich (A3854).

The anti-Nsp3 7G9–1-1 (SARS-CoV-2) monoclonal antibody was generated as described previously (Flaswinkel and Reth, 1994; Yeruva et al., 2020). Briefly, a peptide containing the amino acids DKKIKA-abu-VEEVTTTLEET (orf1a aa pos. 1229–1246; MN908947.2) was synthesized and coupled to OVA (Peps4LS, Heidelberg, Germany). Within this sequence, C was replaced by abu to avoid problems with coupling of the peptide to ovalbumin. C57BL/6 mice were immunized subcutaneously and intraperitoneally with a mixture of 50 µg peptide OVA, 5 nmol CPG oligonucleotide (Tib Molbiol, Berlin, Germany), 100 µl PBS and 100 µl incomplete Freund’s adjuvant. A boost without adjuvant was given six weeks after the primary injection. Fusion was performed according to standard procedures. Hybridoma supernatants were first tested by ELISA. Briefly, supernatants were positively selected for binding to SUD-VE: eBio-DKKIKACVEEVTTTLEET-HHHHHH and negatively selected for binding to SUD-ID: eBio-DKKIKACIDEVTTTLEET-HHHHHHH peptides. In both cases, the respective peptides were bound to avidin-coated ELISA plates from stock. Unbound peptide was removed before hybridoma supernatant was applied. Selected supernatants were further analyzed by Western blot. Anti-Nsp3 7G9–1-1 was cloned based on its ability to bind also in Western blot.

Peroxidase-conjugated secondary anti-mouse (A9917, 1:20,000); and anti-rat (A9037, 1:5,000; A9037) antibodies were obtained from Sigma-Aldrich. Peroxidase-conjugated anti-rabbit secondary antibody (1:1,000) was purchased from DAKO (P0217). Goat anti-rabbit FITC (1:1000; Sigma-Aldrich, F0382) and goat anti-mouse Alexa-555 (1:500; ThermoFisher, A21424) were used as fluorescent secondary antibodies.

The inhibitor ZJ-1, a structurally simplified analog of the natural product OSW-1, was originally designed and synthesized by the research team of Prof. Zhendong Jin at the Department of Pharmaceutical Sciences and Experimental Therapeutics, College of Pharmacy, The University of Iowa, as a novel anticancer agent that exhibits exactly the same potent anticancer activity and therapeutic selectivity as OSW-1 by *in vitro* evaluation (Zhou et al., 2005). Structurally, the C-17 tertiary alcohol in OSW-1 is sterically hindered and is unlikely to be involved in binding to the drug target. By replacing the C-17 hydroxy group with a hydrogen, the synthesis of ZJ-1 (17-deoxy OSW-1) is simpler than that of OSW-1, and the synthetic process developed by Prof. Jin’s team can be readily scaled up. Currently, ZJ-1 is available from Cfm Oskar Tropitzsch GmbH, Germany.

### 2.6 RNA isolation and qPCR

The Bioline ISOLATE II RNA Mini Kit (BIO-52073) was used to isolate RNA from either supernatant or cells according to its standard protocol. For inhibition assays of ZJ-1 with HCoV-OC43, viral RNA isolated from the supernatant was processed by probe-based qPCR (SensiFAST Probe Hi-ROX One-Step Kit (BIO-77005), BIOLINE) using the appropriate primers and probes listed in **Table S1**. For Sybr Green qPCR, total RNA isolated from cells was used as a template to synthesize cDNA using oligonucleotide (dT)18 and SuperScript IV reverse transcriptase (Thermofisher Scientific/Invitrogen, 18090050). Quantification was then performed by SYBR Green qPCR using HA-YFP^C^- and β-actin-specific primers (**Table S1**) and AceQ SYBR qPCR Master Mix (#Q111, Absource/Vazyme).

### 2.7 Luciferase assays and cell viability

For the Renilla luciferase assay, HCoV-229E-RLuc-infected cells (Carbajo-Lozoya et al., 2012) were harvested using the Renilla Luciferase Assay System (Promega, #E2820) according to its standard protocol. Luciferase activity was measured using a CLARIOstar microplate reader (BMG LABTECH). For the Gaussia luciferase assay, the supernatant of MHV-GLuc-infected L929 cells was directly harvested for luciferase activity measurement using the Renilla luciferase substrate. For the NanoBiT luciferase assay, cells from each well in a 96-well plate were lysed with 20 µl NP-40 lysis buffer (1%NP-40, 150 mM NaCl, 50 mM Tris-HCl, pH 8.0) and then treated with 20 µl substrate containing 0.5% Nano-Glo^®^ Luciferase Assay Substrate (Promega, #N113A) in 1x Nano-Glo^®^ Blotting Buffer (Promega, #N242A) for measurement.

Cell viability was measured using the CellTiter-Glo^®^ 2.0 Cell Viability Assay (Promega, #G9242). Cell lines were plated in 96-well plates and incubated with inhibitor concentrations corresponding to each inhibition experiment.

### 2.8 Co-immunoprecipitation and Western blot

HEK293 cells were transfected with plasmids in a 6-well culture plate using Lipofectamine 3000 (Thermofisher). Twenty-four hours after transfection, cells were lysed and processed with GFP-Trap_A bead-based CoIP (ChromoTek) according to its standard protocol. Western blotting protocol was described elsewhere (Carbajo-Lozoya et al., 2014).

### 2.9 Immunofluorescence staining, split-YFP and fluorescence microscopy

The mock and infected cells seeded on coverslips with or without ZJ-1 treatment were washed with 1x PBS (Gibco) and fixed in 4% PFA (Carl Roth GmbH) in PBS for 15 minutes. After a wash step with PBS, cell membranes were permeabilized in 0.1% Triton X-100 (Carl Roth GmbH) in PBS for 10 minutes for 15 minutes. The cells were washed again with PBS and blocked in PBS containing 5% BSA (Carl Roth GmbH) and 0.2% Tween-20 (Sigma-Aldrich Chemie) for one hour at room temperature. The cells were then incubated with primary antibodies of anti-PDI (Invitrogen) at 1:100 or anti-dsRNA (SCICONS) at 1:1000 and anti-OSBP (Sigma-Aldrich) at 1:300 dilution in PBS containing 5% BSA and 0.2% Tween^®^20 at 4°C overnight. After three washes with PBS, cells were incubated with secondary antibodies AlexaFluor^®^555 anti-mouse (Invitrogen) at 1:500 and FITC anti-rabbit (Invitrogen) at 1:1000 in PBS containing 5% goat serum (Bio&SELL GmbH) and 0.2% Tween^®^20 at room temperature in the dark for 1 hour. The cells were then stained with DAPI (Sigma Aldrich) diluted 1:1000 in PBS for 10 minutes in the dark. The cells were washed three times with PBS. Immunostained coverslips were mounted onto glass slides using Roti^®^ Mount-FluorCare (Carl Roth GmbH). Images were captured using a Leica DM4000 B fluorescence microscope with a 63x objective. Split-YFP slides of live cells were prepared as described elsewhere (Ma-Lauer et al., 2016). Here, images were captured using a 40x objective. GFP, RFP, and DAPI or Hoechst images of fixed cells were captured using a 20x objective.

### 2.10 Yeast two hybrid screening

Yeast two-hybrid screening was performed by utilizing a Gateway-compatible yeast 2-hybrid system (Stellberger et al., 2012). First, all SARS2-CoV-2 genes were cloned into pGBKT7g bait vector encoding leucine, and OSBP was cloned into pGADT7g prey vector expressing tryptophan. SARS-CoV-2 subdomains, SUD (a.a. 413–745 in Nsp3), PLP (a.a. 746–1064 in Nsp3), Nsp12 NiRAN (a.a. 1–249 in Nsp12), interface (a.a. 250–396 in Nsp12) and RdRp (a.a. 397–932 in Nsp12), S-RBD (a.a. 325–588 in spike), S2 (a.a. 662–1273 in spike) and S2 Δa.a.1206–1254 (transmembrane domain deleted) were also cloned. The gap in Nsp3 between a.a. 1065–1414 and a.a. 1547–1945 containing transmembrane regions was intentionally excluded. The competent yeast (PJ69–7A) cells transformed with both prey and bait plasmids were cultured in double selection dropout medium (without Leu and Trp) in 96- deep well plates for 4 days and then stamped to both double selection and triple selection dropout agar plates (without Leu, Trp and His) for another 3 days. Theoretically, yeast cells transformed with both prey and bait plasmids should grow on triple selection plates only if the SARS-CoV-2 proteins interact with OSBP. Thus, yeast colonies on double selection plates indicated successful transformation of both prey and bait plasmids and growth on triple selection plates indicated interaction between viral and cellular proteins fused to activation and binding domains of the Gal4 transcription factor. On each agar plate, positive (pGBKT7g-SARS-CoV-SUD plus pGADT7g-RCHY1 and negative controls (pGBKT7g vector plus pGADT7g-OSBP) were included. Conversely, control screenings were performed in the same manner between pGADT7g vector (instead of pGADT7g-OSBP) and bait plasmids fused to all SARS-CoV 2 genes or subdomains.

### 2.11 Statistical analysis

The data from qPCR and luciferase measurements are presented as the mean ± standard deviation of triplicate or quadruplicate values. Statistical analysis is conducted using one-way ANOVA with Dunnett’s multiple comparison test, or student t-test for two-group analysis utilizing GraphPad Prism 10 software with a significance level set at α=0.05. Graphs for all data are created using GraphPad Prism 10, using both the raw data and statistical analyses performed within the same software. The symbols in charts denote the following significance levels of p-values: “ns” (not significant) indicates p>=0.05, one asterisk (*): 0.01<p<0.05, two asterisks (**): 0.001<p<0.01, three asterisks (***): p<0.001, four asterisks (****): p<0.0001.

## 3 Results

### 3.1 OSBP positively regulates viral replication of HCoV-229E-RLuc

**Figure 1:**
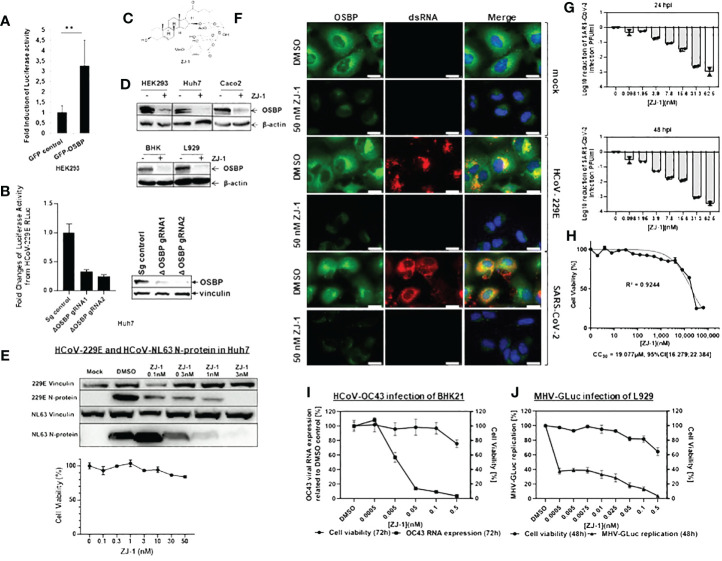
OSBP regulates and OSBP-binding compound ZJ-1 inhibits coronavirus replication. **(A)** HEK293 were grown in 96-well plates and transfected with plasmid pCG1-APN together with pDEST-GFP control or pDEST-GFP-OSBP. 24 hours after transfection, cells were infected with HCoV-229E-RLuc (MOI=0.1). After further 24 hours, cells were lysed for luciferase activity measurement (n=6). **: p<0.01. **(B)** For the luciferase activity assay, small guide RNA (sg RNA) control as well as OSBP knockout (ΔOSBP) cell pools seeded in a 96-well plate with similar cell numbers were infected with HCoV-229E-RLuc (MOI=6). Twenty-four hours later, the cells were lysed for measurement of Renilla luciferase activity. The experiment was performed in six biological repeats. For the western blot assay, the sg control as well as the OSBP knockout cell pools, seeded at similar cell numbers in a 6-well plate, were lysed with NP-40 lysis buffer and processed for Western blot using anti-OSBP and anti-vinculin antibodies. **(C)** Chemical structure of ZJ-1 (17-dehydroxy OSW-1). **(D)** HEK293, Huh7, and Caco-2 cells were treated with DMSO or 50 nM ZJ-1 for 24 hours. BHK cells were treated with DMSO or 500 pM ZJ-1 for 72 hours. L929 cells were treated with DMSO or 50 pM ZJ-1 for 48 hours. Cells were then lysed with NP-40 lysis buffer and processed for Western blotting using anti-OSBP and anti-β-actin (loading control) antibodies. **(E)** Western blot analysis of N protein expression in Huh7 cells infected with HCoV-229E- and HCoV-NL63. Cells were treated at increasing concentrations of ZJ-1 for 24 hours and then processed for WB analysis. As a measure of viral replication N-protein was detected with a mouse anti-N mab cross-reactive for both viruses. An anti-vinculin antibody was used as a loading control. 229E Vinculin/229E N-protein and NL63 Vinculin/NL63 N-protein indicate that the same protein samples of the respective infection experiments were used. For the cell viability assay, Huh7 cells seeded in a 96-well plate were treated with ZJ-1 at different concentrations as indicated in the graph. Twenty-four hours later, cell viability was measured (n=3). **(F)** Huh7 cells were infected with HCoV-229E (MOI=1) and SARS-CoV-2 (MOI=0.1; B.1, strain EPI_ISL_406862) and treated with either DMSO or 50 nM ZJ-1 for 24 h before fixation. Anti-OSBP and anti-dsRNA antibodies were used for immunofluorescence staining. The scale bar represents 25 µm. **(G)** Caco-2 cells were infected with SARS-CoV-2 (MOI=0.1) and treated with ZJ-1 for 24h or 48h, respectively. The supernatants were then plaque titrated and the Log10 reduction of PFU/ml of the supernatants was calculated. **(H)** In parallel to SARS-CoV-2 infection, cell viability under ZJ-1 treatment was measured in Caco-2 cells at 72h. **(I)** BHK cells were infected with HCoV-OC43 (MOI 0.01) and then treated with ZJ-1 for 72 h before viral RNA was isolated from the supernatant for qPCR analysis (n=3). Cell viability was measured by CellTiterGlo (Promega) after 72 h (n=4). **(J)** L929 cells were infected with MHV-GLuc (MOI 0.01) and then treated with ZJ-1 for 48 h. Subsequently, Gaussia luciferase activity was then measured from the supernatant (n=3). Cell viability after 48 hours of inhibitor treatment in L-929 cells was measured with CellTiterGlo (n=3).

It has been shown that knockdown of OSBP impairs the replication of HCV and Aichi virus (Amako et al., 2009; Ishikawa-Sasaki et al., 2018), suggesting an auxiliary function of OSBP in the replication of (+) ssRNA viruses. To determine whether OSBP also mediates coronavirus growth, HEK293 cells transiently expressing the HCoV-229E aminopeptidase N (APN) receptor and GFP-OSBP or GFP control were challenged with HCoV-229E carrying a Renilla luciferase reporter (HCoV-229E-RLuc) (Ma-Lauer et al., 2020). As a result, expression of GFP-OSBP resulted in significantly increased luciferase activity of HCoV-229E-RLuc compared to the GFP control (**Figure 1A**). This suggests that OSBP positively mediates HCoV-229E replication.

To confirm that OSBP enhances coronavirus replication, an OSBP knockout was generated in Huh7 cells, which can be naturally infected by several human coronaviruses. Subsequently, OSBP knockout Huh7 cell pools with two different CRISPR guide RNAs (gRNA) were challenged with HCoV-229E-RLuc. As shown in **Figure 1B**, luciferase activity carried by HCoV-229E-RLuc was significantly reduced in both independent OSBP knockout Huh7 cell pools. Western blot showed that the knockout of OSBP was effective in both pools, although not 100% efficient (**Figure 1B**). Taken together, OSBP positively regulates HCoV-229E-RLuc replication.

### 3.2 OSBP binding compound ZJ-1 inhibits replication of coronaviruses

We further investigated whether an OSBP-binding compound could inhibit coronavirus growth, as OSBP has stimulated HCoV-229 replication and OSBP-binding compounds have been reported to have antiviral activity against enteroviruses and HCV (Amako et al., 2009; Albulescu et al., 2015; Strating et al., 2015; Albulescu et al., 2017; Roberts et al., 2019). To achieve this goal, a novel OSBP-binding compound, ZJ-1 (**Figure 1C**), was applied to the SARS-CoV-2 replication assay. ZJ-1 is a structurally simplified analog of the oxysterol binding protein (OSBP)-binding compound OSW-1. Similar to the effect of its parent compound OSW-1 (Roberts et al., 2019), treatment with ZJ-1 also reduces endogenous OSBP in several cell lines (**Figure 1D**). Western blot measuring nucleocapsid expression showed that ZJ-1 treatment reduced protein intensity of nucleocapsid of HCoV-229E and HCoV-NL63 in Huh7 cells, while cell viability assay revealed low cytotoxicity of ZJ-1 to Huh7 cells (**Figure 1E**).

In addition, immunofluorescence microscopy using anti-OSBP and anti-dsRNA antibodies suggested antiviral activity of ZJ-1 against SARS-CoV-2 and close association of OSBP with replication organelles (**Figure 1F**). In DMSO-treated mock-infected Huh7 cells, endogenous OSBP is distributed in the cytosol but concentrated in the ER (**Figure 1F**; **Supplementary Figure S1**), which is consistent with previous reports (Mesmin et al., 2013; Albulescu et al., 2017). dsRNA was not detectable as there was no viral infection (**Figure 1F**). In ZJ-1-treated mock-infected cells, the protein level of OSBP is significantly reduced (**Figure 1F**; **Supplementary Figure S1**), which is consistent with the Western blot results (**Figure 1D**). In the DMSO-treated HCoV-229E-infected cells, OSBP clearly co-localizes with dsRNA (**Figure 1F**, images in the 3rd row), indicating its close proximity to replication organelles (DMVs). In DMSO-treated SARS-CoV-2 infected cells, the distribution of both dsRNA and OSBP is slightly different, although they still co-localize (**Figure 1F**, images on the 5th row). Here, dsRNA is distributed almost throughout the cytosol and weak OSBP signal is also detected in the nucleus for an unknown reason. Notably, both HCoV-229E and SARS-CoV-2 replication were severely impaired by ZJ-1 treatment, as dsRNA was almost undetectable in ZJ-1-treated virus-infected Huh7 cells (**Figure 1F**, images in the 4th and 6th rows), suggesting antiviral activity of ZJ-1 not only for HCoV-229E but also for SARS-CoV-2.

To test whether the antiviral effect of ZJ-1 against SARS-CoV-2 is Huh7 specific, Caco-2 cells, which are much more susceptible to infection by SARS-CoV-2, were used (Saccon et al., 2021). Plaque titration experiments from supernatants of Caco-2 cells treated with ZJ-1 showed that nanomolar range of ZJ-1 treatment (62.5 nM) resulted in approximately three log reduction of viral replication at both 24 and 48 h p.i. (**Figure 1G**), while the 72 h ZJ-1 CC_50_ in Caco-2 cells was found to be 19.1 µM (**Figure 1H**). This result demonstrates that ZJ-1 inhibits SARS-CoV-2 replication in different cell types.

It was further investigated whether ZJ-1 also inhibited the viral growth of HCoV-OC43 and murine hepatitis virus (MHV) in BHK21 and L929 cells, respectively. The qPCR assay measuring OC43 viral RNA (**Figure 1I**) and the luciferase assay measuring the activity of the Gaussia luciferase reporter carried by MHV-Gaussia luciferase (MHV-GLuc) (**Figure 1J**) showed that both viruses were also inhibited by ZJ-1.

### 3.3 SARS-CoV-2 viral proteins involved in DMV formation target OSBP

**Figure 2:**
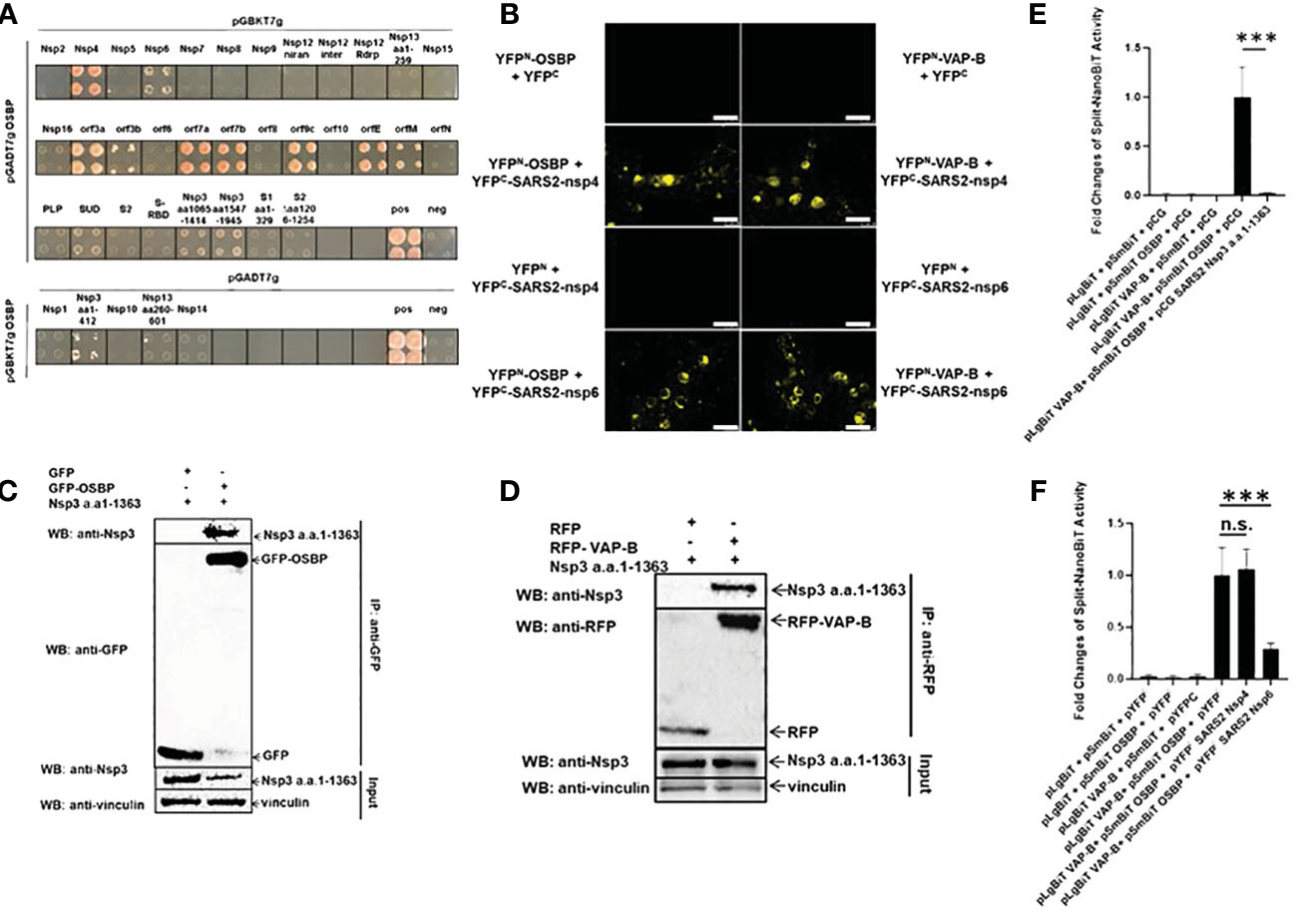
SARS-CoV-2 Nsp3, Nsp4 and Nsp6 fragments target OSBP and VAP-B. **(A)** Yeast strain PJ69–7A cells transformed with both prey and bait plasmids could only grow on a triple selection plate if the SARS-CoV-2 (SARS2) protein encoded by the bait plasmid interacted with OSBP encoded by the prey plasmid. Since SARS2-Nsp1, Nsp3 (aa1–412), Nsp10, Nsp13 (aa260–601) and Nsp14 expressed in the pGBKT7g bait vector are not specifically linked to the prey vector, the interaction of these genes with OSBP was performed with exchanged vectors. This figure shows the growth of yeast on triple selection agar plates, indicating protein-protein interactions. In total, Nsp3 a.a.1–412, Nsp4, nsp6, orf3a, orf3b, orf7a, orf7b, orf9c, orfE and orfM interact with OSBP by yeast two hybrid screening. **(B)** HEK293 cells grown on coverslips in 24-well plates were transfected with pDEST plasmids expressing YFP^N^-OSBP, YFP^N^-VAP-B, YFP^C^-SARS2-Nsp4/-Nsp6, YFP^C^, YFP^N^. Images of live cells were taken 24 hours after transfection using a Leica DM4000 B fluorescence microscope. Scale bar represents 25 µm. **(C)** Plasmids expressing SARS-CoV-2-Nsp3 a.a.1–1363, GFP or GFP-OSBP as indicated were transfected into HEK 293 cells in 10 cm dishes. Cells were harvested 24 h post-transfection for a GFP trap-based pulldown assay. **(D)** Plasmids constructed as pCG-SARS2-Nsp3 a.a.1–1363, pDEST-RFP or pDEST-RFP-VAPB were co-transfected into HEK293 cells in a 6-well plate. 24 h after transfection, cells were lysed and purified by RFP trapping (Chromotek). Input and immunoprecipitation (IP) samples were analyzed by Western blotting with anti-RFP, anti-SARS-CoV-2 Nsp3 or anti-vinculin antibodies. **(E, F)** The split-NanoBiT plasmids indicated in the figures were transfected into HEK293 cells in a 96-well plate using Lipofectamine 3000. Twenty-four hours after transfection, cells were lysed for measurement of nano-luciferase activity (n=6). n.s.: not significant; p>=0.05, ***: p<0.001.

Overexpressing (**Figure 1A**) and knocking out (**Figure 1B**) OSBP tends to stimulate and reduce coronavirus replication, respectively. In addition, ZJ-1, which reduces endogenous OSBP, inhibits viral replication (**Figures 1D–J**). Therefore, OSBP may be an auxiliary host factor for coronaviruses. To further investigate how viral proteins use OSBP to promote viral growth, OSBP was used as a bait to screen SARS-CoV-2 viral proteins for viral-host interactions by yeast 2-hybrid (Y2H) assay. As a result, SARS-CoV-2 nonstructural protein 4 (Nsp4), Nsp6, Nsp3 a.a.1–412, envelope protein (orfE), membrane protein (orfM), and accessory proteins orf3a, orf3b, orf7a, orf7b, and orf9c were shown to be binding partners of OSBP (**Figure 2A**). The Nsp3 SUD domain, Nsp3 a.a.1065–1414, and Nsp3 a.a.1547–1945 also appear to interact weakly with OSBP (**Figure 2A**). The full-length Nsp3 protein was not tested in the Y2H assay because it was too large to be successfully cloned into the yeast vectors. The various negative controls are shown in **Supplementary Figure S2**.

Among the coronaviral proteins, the non-structural proteins Nsp3, Nsp4, and Nsp6 are involved in DMV formation (Angelini et al., 2013; Lundin et al., 2014; Wolff et al., 2020). In the Y2H assay, Nsp3 fragments, Nsp4 and Nsp6 appeared as interacting proteins of OSBP (**Figure 2A**). OSBP is known to mediate the exchange of cholesterol and PI4P between the membrane contacts of the ER and DMVs (Nagy et al., 2016). Therefore, it was necessary to examine the binding of OSBP to Nsp4 and Nsp6 in human cells and to investigate whether OSBP interacts with full-length Nsp3.

The ER-localized vesicle-associated membrane protein (VAMP)-associated proteins (VAPs), including VAP-A and VAP-B, anchor OSBP to the ER membrane (Furuita et al., 2010; Baron et al., 2014) and are targeted by some viral proteins (Gao et al., 2004; Hamamoto et al., 2005). Therefore, it was also interesting to analyze the binding between one of the VAPs and the viral proteins involved in DMV formation. OSBP, VAP-B, SARS-CoV-2 Nsp4 and Nsp6 were cloned into split-YFP vectors (Lei et al., 2021) to study their binding and to visualize the binding sites. Nsp3 could not be cloned into split-YFP vectors due to its large size. The split-YFP assay in HEK293 cells indicates that Nsp4 and Nsp6 interact with both OSBP and VAP-B in the cytosol with accumulation at ER-like structures (**Figure 2B**).

Since the full-length Nsp3 always proved to be too large for successful cloning, a large fragment of Nsp3 (a.a.1–1363), covering 70% of the full-length ORF, was alternatively examined for interaction with OSBP in human cells. The plasmid pCG-SARS-CoV-2 Nsp3 a.a.1–1363 was co-transfected with either pDEST-GFP control or pDEST-GFP-OSBP into HEK293 cells for a GFP-trap based co-immunoprecipitation (CoIP) assay. As shown in **Figure 2C**, SARS-CoV-2 Nsp3 a.a.1–1363 could be co-immunoprecipitated with GFP-OSBP but not with GFP control, indicating the affinity of OSBP for this Nsp3 fragment. In addition, Nsp3 a.a.1–1363 also showed binding activity to VAP-B (**Figure 2D**). Thus, all SARS-CoV-2 viral proteins involved in DMV formation (Nsp4, Nsp6, and Nsp3) target OSBP, which mediates cholesterol transfer from the ER to DMVs, and VAP-B, which is known to anchor OSBP to the ER membrane.

### 3.4 SARS-CoV-2 Nsp3 a.a.1–1363 completely blocks and Nsp6 impairs binding between OSBP and VAP-B

OSBP has previously been reported to interact with VAP-B (Baron et al., 2014). In addition, both proteins are targeted by viral Nsp3, Nsp4 and Nsp6 (**Figures 2B–D**). Therefore, it was also interesting to investigate whether the viral proteins could enhance or disrupt the binding between VAP-B and OSBP. For this purpose, VAP-B and OSBP were cloned into the split-NanoBiT vector p1.1N LgBiT (large BiT) and p2.1N SmBiT (small BiT), respectively. The plasmids expressing LgBiT-VAP-B and SmBiT-OSBP fusion were then co-transfected into HEK293 cells for measurement of split-NanoBiT luciferase activity in the absence or presence of viral proteins. As shown in **Figure 2E**, a clear interaction was detected when both LgBiT-VAP-B and SmBiT-OSBP were co-transfected into cells in the absence of SARS-CoV-2 Nsp3 a.a.1–1363. However, in the presence of SARS-CoV-2 Nsp3 a.a.1–1363, the split-NanoBiT signal resulting from the interaction between LgBiT-VAP-B and SmBiT-OSBP was almost completely abolished. Unlike the large Nsp3 fragment, Nsp4 does not alter the binding between VAP-B and OSBP, whereas Nsp6 seems to interfere with the binding signal to some extent (**Figure 2F**). It was then investigated whether Nsp3 a.a.1–1363 and Nsp6 could down-regulate the HSV-TK promoter carried by the split-NanoBiT vectors to cause a reduced split-NanoBiT signal. For this purpose, GFP was cloned into a split-NanoBiT vector generating a SmBiT-GFP fusion construct under the control of the HSV TK promoter. This plasmid expressing SmBiT-GFP was co-transfected with control or viral protein expressing vectors. As shown in **Supplementary Figure S3**, none of the SARS-CoV-2 Nsp3 large fragment, Nsp4, and Nsp6 downregulated the HSV TK promoter, as the SmBiT-GFP signal intensity was comparable in all samples, excluding the influences of the Nsps on the TK promoter.

### 3.5 OSBP stabilizes SARS-CoV-2 accessory proteins orf7a, orf7b, and orf3a

**Figure 3:**
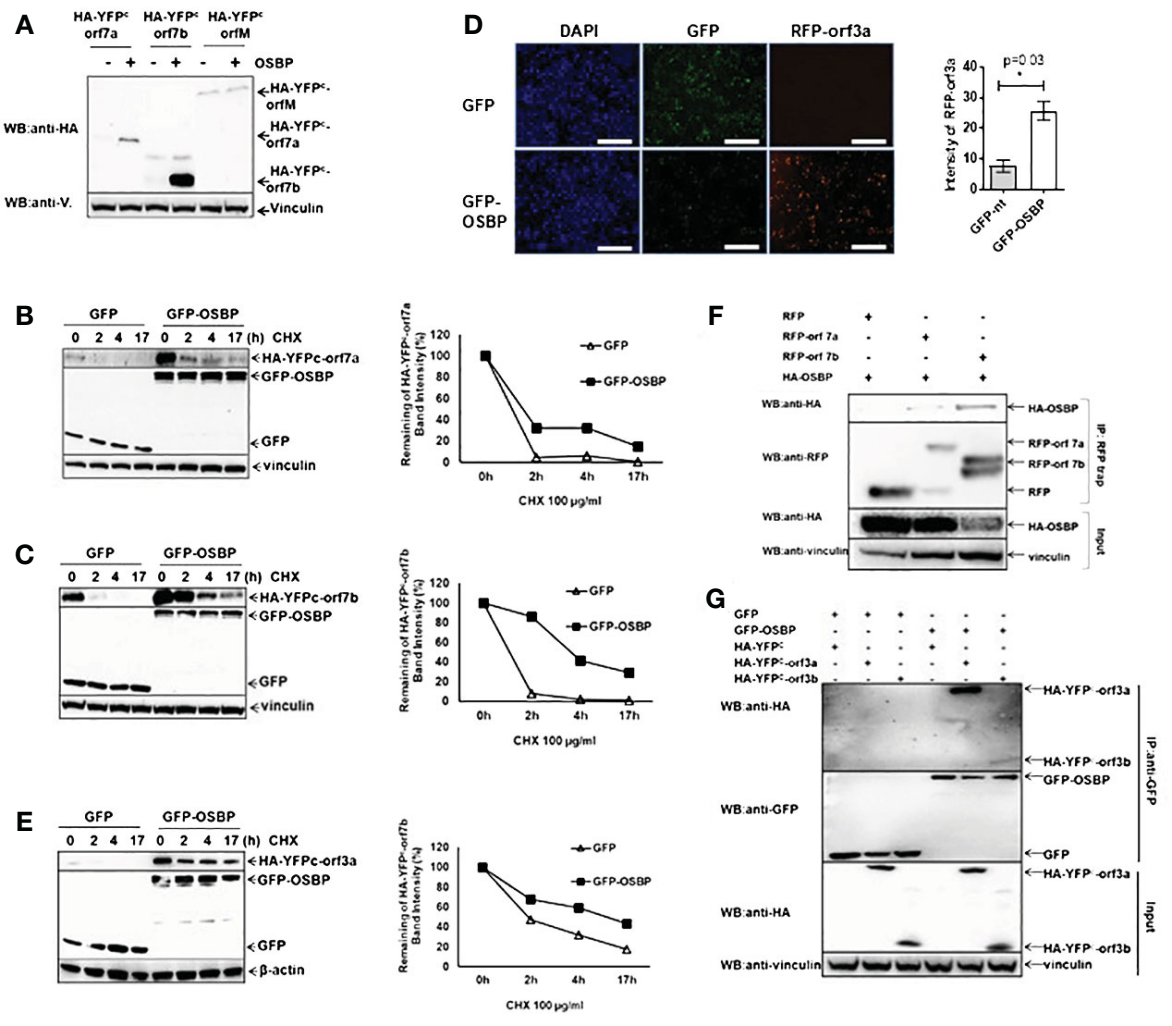
OSBP stabilizes the SARS-CoV-2 accessory proteins orf7a, orf7b and orf3a. **(A)** HEK293 cells grown in a 12-well plate were transfected with pDEST-HA-YFPC -orf7a/-orf7b/-orfM together with either pDEST-GFP control or pDEST-GFP-OSBP plasmid. Cells were harvested for Western blotting 24 hours after transfection. **(B, C, E)** The indicated plasmids were transfected into HEK293 cells in 24-well plates. Twenty-four hours after transfection, cells were treated with CHX at a final concentration of 100 µg/ml. After treatment with CHX, cells were harvested at different time points as indicated in the graphs. **(D)** The indicated plasmids were co-transfected into HEK293 cells in a 24-well plate using Lipofectamine 3000. Twenty-four hours after transfection, cells were fixed and stained with DAPI. Images were captured using an EVOS M7000 fluorescence microscope (20x objective; Thermofisher). Celleste software was used to analyze the RFP intensity of three randomly taken images from each treatment. Scale bar represents 25 µm. *: p<0.05. **(F)** Plasmids were constructed as pDest-HA-OSBP, pDest-RFP and pDest-RFP-orf7a/orf7b. HEK293 cells were co-transfected with the indicated plasmids in 6-well plates using Lipofectamine. Cells were harvested 24 hours post-transfection for GFP trap-based pulldown assays. **(G)** Plasmids were constructed as pDest-GFP, pDest-GFP-OSBP, pDest-HA-YFP^C^ and pDest-HA-YFP^C^-orf3a or -orf3b. HEK293 cells were co-transfected with the indicated plasmids in 6-well plates using Lipofectamine. Cells were harvested 24 hours post-transfection for a GFP-trap based immunoprecipitation assay.

According to the Y2H screening results, OSBP should also interact with SARS-CoV-2 orf7b, orf7a, orfM, orfE, orf3a, orf3b, and orf9c (**Figure 2A**). The original idea was to confirm the interaction between OSBP and these viral proteins in human cells. To achieve this goal, SARS-CoV-2 orf7b was first cloned into the pDEST-HA tag vector to generate the HA-orf7b construct under the control of the CMV promoter for further co-immunoprecipitation (CoIP) assays. However, for an unknown reason, despite the correct nucleotide sequences, there was no expression of HA-orf7b. Therefore, SARS-CoV-2 orf7b was alternatively cloned into a split YFP vector pDEST-HA-YFP^C^ (Lei et al., 2021) to generate a pDEST-HA-YFP^C^-orf7b construct. YFP^C^ consists of the C-terminal fragment of YFP (a.a. 156–239). Identical amounts of plasmid DNA were transfected into HEK293 cells for CoIP assay. Interestingly, a strong increase in HA-YFP^C^-orf7b protein accumulation was observed in the presence of OSBP (**Supplementary Figure S4**, lane 2, input) compared to the control (**Supplementary Figure S4**, lane 1). Although HA-YFP^C^-orf7b was successfully co-immunoprecipitated by GFP-OSBP (lane 2), a firm conclusion from this assay for the binding of OSBP to orf7b cannot be drawn from this assay due to the strong increase in orf7b expression in the presence of OSBP. The size of the HA-YFP^C^ control protein is too small to be detected by Western blot (lane 3). A further Western blot confirmed that OSBP affects the protein level of SARS-CoV-2 orf7b (**Figure 3A**). Orf7a and orfM were used as random controls. By chance it was found that OSBP stimulated protein accumulation of orf7a, but it showed no effect on orfM. Since orf7a, orf7b, and orfM were cloned into the same plasmid pDEST‐HA‐YFP^C^ under the control of the CMV promoter, we expected that the OSBP-mediated protein accumulation of orf7a and orf7b was a result of the protein level rather than the transcription level. This assumption was confirmed by qPCR analysis (**Supplementary Figure S5**). To determine whether the protein accumulation of orf7a and orf7b was due to increased protein biosynthesis or decreased protein degradation, cycloheximide (CHX) chase assays were performed to prevent protein synthesis and determine protein stability. As shown in **Figures 3B, C**, co-expression of OSBP resulted in prolonged half-lives of orf7a and orf7b, respectively. Thus, OSBP stabilizes SARS-CoV-2 orf7a and orf7b at the protein level.

Whether OSBP could stabilize the other binding partners was also tested. pDEST plasmids expressing RFP-fused viral proteins under the control of the CMV promoter were co-transfected with either pDEST-GFP or pDEST-GFP-OSBP vectors into HEK293 cells. The transfected cells were observed 24 hours after transfection. The results showed that OSBP also increased the protein level of orf3a (**Figure 3D**), but had no effect on Nsp4, Nsp6, orfE, orf3b and orf9c (**Supplementary Figure S6**). In addition, the protein level of Nsp3 a.a.1–1363 was not altered by OSBP, as shown by Western blot (**Supplementary Figure S6**). The CHX-chase assay showed that orf3a can also be stabilized by OSBP (**Figure 3E**).

Orf7a, orf7b and orf3a are binding candidates of OSBP according to the Y2H screening (**Figure 2A**) and they can be stabilized by OSBP (**Figures 3A–E**). Therefore, it is necessary to investigate their interactions in human cells. CoIP results confirmed the interactions (**Figures 3F, G**), suggesting that these accessory proteins are stabilized by OSBP probably through direct interaction. In addition, OSBP also appears to bind orf3b (**Figure 3G**), which is consistent with the Y2H screening result (**Figure 2A**).

## 4 Discussion

OSBP mediates the shuttling of cholesterol/PI4P between the ER and DMVs in cells infected with some (+) ssRNA viruses, leading to the accumulation of cholesterol in DMV membranes, which is required for the morphogenesis of replication organelles (Nagy et al., 2016). In this study, OSBP overexpression stimulates and OSBP knockout reduces HCoV-229E-RLuc viral replication. Thus, both in theory and in practice, OSBP should be a positive host regulator of coronavirus replication. The OSBP-binding compound ZJ-1 inhibits the replication of several coronaviruses, presumably also due to its ability to strongly deplete endogenous OSBP.

VAP-A and VAP-B recruit OSBP to the ER membrane by binding to the FFAT motif of OSBP (Furuita et al., 2010; Baron et al., 2014). Both OSBP and VAP-B target SARS-CoV-2 Nsp3, Nsp4, and Nsp6, which are involved in DMV formation (Angelini et al., 2013; Lundin et al., 2014; Wolff et al., 2020). Therefore, it is not surprising that OSBP co-localizes with dsRNA, indicating the location of viral RNA replication in coronavirus-infected cells. In addition, OSBP interacts with and increases the protein levels of the SARS-CoV-2 accessory proteins orf3a, orf7a, and orf7b. It is speculated that orf3a, orf7a, and orf7b are stabilized by binding to OSBP. Most interestingly, SARS-CoV-2 orf3a, orf7a, and orf7b are also all interacting candidates of VAP-A and VAP-B (Laurent et al., 2020; St-Germain et al., 2020; Liu et al., 2021). It is likely that these viral accessory proteins also play a role in supporting OSBP-mediated lipid transfer from the ER to the DMVs.

The essential non-structural coronavirus proteins are much more conserved than the partially dispensable accessory proteins. Since the host auxiliary factor OSBP, which mediates lipid transfer from the ER to the DMVs, positively regulates replication of several coronaviruses, targeting of OSBP by Nsp3, Nsp4, and Nsp6 may be common and important for replication of all coronaviruses. Nsp3, Nsp4, and Nsp6 probably interact with OSBP and VAPB to localize DMVs to the ER to form membrane contact sites between these two organelles, similar to the function of binding between VAPs and HCV NS5A and NS5B (Gao et al., 2004; Hamamoto et al., 2005). MHV Nsp3 has been shown to be a direct component of pore complexes embedded in DMV membranes in MHV-infected cells (Wolff et al., 2020). Thus, binding of DMV membrane-embedded Nsp3 to ER membrane-localized VAP-B may anchor DMVs to the ER and lead to intimate membrane contact sites. Although the direct interaction between OSBP and VAP-B is disrupted by Nsp3, Nsp3 binds OSBP on the one hand and VAP-B on the other, so that OSBP is still recruited adjacent to the ER to mediate lipid transfer from the ER to DMVs (**Supplementary Figure S7**). According to our Y2H results, the N-terminal region of Nsp3 should be mainly responsible for the interaction with OSBP. However, it remains unclear which region of OSBP is actually critical for binding to Nsp3. However, the FFAT motif of OSBP interacts with VAPs (Furuita et al., 2010; Baron et al., 2014) and the binding of OSBP to VAP-B can be abolished by Nsp3. Therefore, Nsp3 may also interact with a region of OSBP that includes the FFAT motif.

It has been previously published that OSBP-binding compounds exert antiviral activity. For example, OSW-1 is a natural compound extracted from Ornithogalum saundersiae (Burgett et al., 2011). It exhibits potent anticancer activity and efficiently inhibits the replication of enteroviruses (Albulescu et al., 2015). ITZ is another anticancer agent that targets OSBP. ITZ inhibits OSBP-mediated cholesterol/PI4P exchange and viral replication of poliovirus and HCV (Strating et al., 2015). The compound TTP-8307 is also an OSBP inhibitor with antiviral activity, suppressing viral replication of enteroviruses, EMCV and HCV (Albulescu et al., 2017). Similar to ITZ, TTP-8307 interferes with OSBP-mediated lipid transfer. However, unlike ITZ, TTP-8307 disrupts the binding between the FFAT motif of OSBP and VAP-A, which recruits OSBP to the ER membrane (Furuita et al., 2010; Albulescu et al., 2017). Thus, there is no doubt that OSBP is a potential target for broad-spectrum antiviral drugs. However, all these known OSBP-binding compounds either decrease OSBP protein levels, such as OSW-1 (Roberts et al., 2019) and ZJ-1, or inhibit the cellular function of OSBP as a lipid transporter that also mediates the shuttling of cholesterol between the Golgi apparatus and the ER, which is required for host cells. It can be speculated that the ZJ-1/OSBP complex has a higher affinity for ubiquitin ligases, thus reducing OSBP. However, the mechanism by which ZJ-1 reduces OSBP is not known. Therefore, their cytotoxicity as drug candidates requires special attention. For SARS-CoV-2, the ideal compounds would be molecules that disrupt the binding between OSBP and Nsp3 or even the formation of viral protein complexes with OSBP and VAP-B, without greatly reducing cellular OSBP or inhibiting cellular functions of OSBP.

## Data availability statement

The raw data supporting the conclusions of this article will be made available by the authors, without undue reservation.

## Ethics statement

Ethical approval was not required for the studies on humans in accordance with the local legislation and institutional requirements because only commercially available established cell lines were used. Ethical approval was not required for the studies on animals in accordance with the local legislation and institutional requirements because only commercially available established cell lines were used.

## Author contributions

YM-L: Visualization, Validation, Writing – review & editing, Writing – original draft, Methodology, Investigation, Formal analysis, Data curation, Conceptualization. PL: Writing – review & editing, Validation, Methodology, Investigation, Data curation. DN: Resources, Writing – review & editing, Investigation. ARi: Methodology, Data curation, Writing – review & editing, Investigation. KP: Formal analysis, Writing – review & editing, Methodology, Investigation, Data curation. BB: Writing – review & editing, Methodology, Investigation. YR: Writing – review & editing, Methodology, Investigation. CX: Writing – review & editing, Methodology, Investigation. SS: Data curation, Writing – review & editing, Methodology, Investigation. JL: Writing – review & editing, Methodology, Investigation. PB: Writing – review & editing, Methodology, Investigation. EB: Writing – review & editing, Methodology, Investigation. HQ: Resources, Writing – review & editing. ARo: Writing – review & editing, Resources. EK: Methodology, Investigation, Writing – review & editing, Resources. HF: Writing – review & editing, Resources, Methodology, Investigation. CD: Writing – review & editing, Supervision, Resources, Methodology. ZJ: Writing – review & editing, Resources. AB: Writing – review & editing, Resources, Writing – original draft, Supervision, Project administration, Methodology, Investigation, Funding acquisition, Formal analysis, Data curation, Conceptualization.

## Supplementary material

Supplementary Figure 1 Co-localization of OSBP with the ER marker PDI. Huh7 cells were treated with either DMSO or 50 nM ZJ-1/DMSO for 24 hours before fixation, and then immunofluorescent staining was performed using anti-OSBP and anti-PDI antibodies. Scale bar represents 25 µm.

Supplementary Figure 2 Negative controls used in the yeast 2-hybrid assay. **(A, B)** display the results of the yeast 2-hybrid control screening using the pGADT7g empty prey vector instead of pGADT7g-OSBP, together with bait plasmids fused with all SARS-CoV 2 genes or fragments. **(A)** represents the successful co-transformation of all the prey and bait vectors under double selection (-Trp, -Leu). **(B)** shows the result of the triple selection (-Trp, -Leu, -His), indicating protein-protein interaction. Since SARS2-Nsp1, Nsp3 (a.a.1–412), Nsp10, Nsp13 (a.a.260–601), and Nsp14 expressed in pGBKT7g bait vector unspecifically bound to the empty prey vector, the experiment of those genes was performed in an exchanged way. **Supplementary Figure S2C** displays the growth of yeast on a double selection plate in the OSBP yeast 2-hybrid screening assay, parallel to .

Supplementary Figure 3 SARS-CoV-2 Nsp3 a.a.1–1363, Nsp4, and Nsp6 do not downregulate the HSV TK promoter carried by split-NanoBiT vectors. The indicated plasmids were co-transfected into HEK293 cells in a 24-well plate using Lipofectamine 3000. After 24 hours, the cells were fixed with 4% PFA and stained with Hoechst 33342 (Invitrogen). The intensity of GFP was quantified using ImageJ software. The graphs present mean values and standard deviations calculated from four randomly selected pictures of each sample. Scale bar represents 150 µm.

Supplementary Figure 4 OSBP enhances the protein level of SARS-CoV-2 orf7b. Plasmids expressing the indicated constructs pDEST-HA-YFP^C^-orf7b, pDEST-GFP-OSBP, and their respective controls were transfected into HEK293 cells in a 6-well plate format. After 24 hours, cells were harvested for a GFP-trap-based pulldown assay.

Supplementary Figure 5 OSBP does not regulate SARS-CoV-2 orf7a and orf7b at the mRNA level. HEK293 cells were transfected with pDEST-HA-YFP^C^-orf7a/-orf7b and either pDEST-GFP control or pDEST-GFP-OSBP plasmid in a 24-well plate. After 24 hours, cellular RNA was isolated for cDNA synthesis and SYBR Green qPCR. The relative YFP^C^ mRNA was calculated as the ratio of YFP^C^ mRNA to its corresponding β-actin mRNA in each sample. Statistical analysis based on biological triplicates showed no significant difference between the GFP control group and the GFP-OSBP group for both relative YFP^C^-orf7a mRNA and YFP^C^-orf7b mRNA. The abbreviation ‘n.s.’ was used to indicate that the difference was not significant.

Supplementary Figure 6 OSBP does not affect the protein levels of SARS-CoV-2 Nsp3 a.a.1–1363, Nsp4, Nsp6, orf3b, orf9c, and orfE. Plasmids were constructed as pCG-SARS2-Nsp3 a.a.1–1363, pDest-HA or pDest-HA-OSBP for the Western blot. HEK293 cells were co-transfected with the indicated plasmids in a 12 well-plate using Lipofectamine 3000 and incubated overnight. The protein samples were analyzed with western blot using anti-HA, anti-Nsp3, or anti-vinculin antibodies. Fluorescence microscopy was performed by co-transfecting the indicated plasmids into HEK293 cells in a 24-well plate using Lipofectamine 3000. After 24 hours, cells were fixed and stained with DAPI. Pictures were taken using an EVOS M7000 fluorescence microscope (Thermofisher, 20x objective) and RFP intensity was analyzed using Celleste software on three randomly selected images from each treatment. Scale bar represents 150 µm.

Supplementary Figure 7 Illustration of the process of OSBP-mediated lipid transfer with and without coronavirus infection. In the absence of viral infection, OSBP is anchored by VAPB located in the ER, which regulates the exchange of cholesterol/PI4P between the ER and Golgi apparatus (Mesmin et al., 2013). However, in the presence of coronavirus infection, the interaction between VAPB and OSBP is disrupted by coronaviral Nsp3 as demonstrated in . Nsp3, which is embedded in the DMV membrane (Wolff et al., 2020), interacts with VAPB () to form intimate membrane contact sites between DMVs and ER. Furthermore, Nsp3 interacts with OSBP () to recruit it next to the endoplasmic reticulum (ER) and facilitate lipid transfer from the ER to double-membrane vesicles (DMVs).

Supplementary Table 1 Primer list.
