# Supplementary figures and images for: Oxysterole-binding protein targeted by SARS-CoV-2 viral proteins regulates coronavirus replication
Source: Front Cell Infect Microbiol. 2024 Jul 25;14:1383917. doi: 10.3389/fcimb.2024.1383917 (PMC11306179; doi:10.3389/fcimb.2024.1383917)

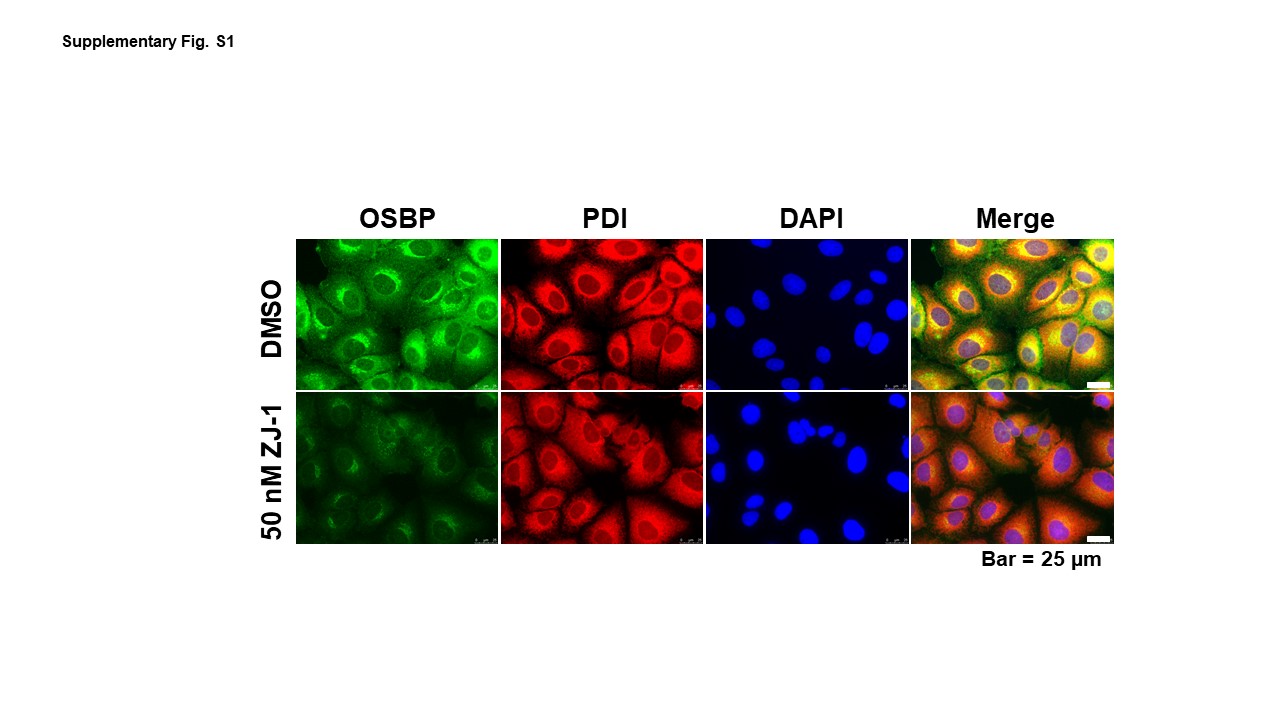

Supplement: Supplementary Figure 1 — Co-localization of OSBP with the ER marker PDI. Huh7 cells were treated with either DMSO or 50 nM ZJ-1/DMSO for 24 hours before fixation, and then immunofluorescent staining was performed using anti-OSBP and anti-PDI antibodies. Scale bar represents 25 µm. [file Image_1.jpg]

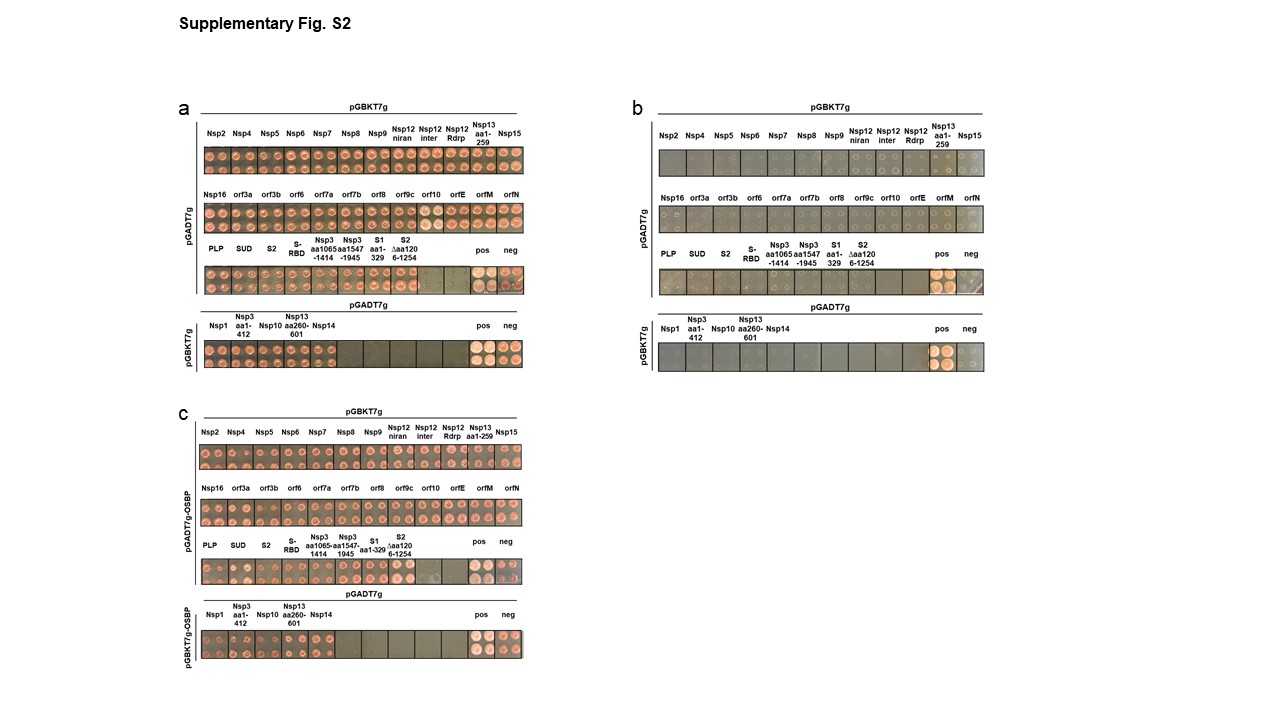

Supplement: Supplementary Figure 2 — Negative controls used in the yeast 2-hybrid assay. (A, B) display the results of the yeast 2-hybrid control screening using the pGADT7g empty prey vector instead of pGADT7g-OSBP, together with bait plasmids fused with all SARS-CoV 2 genes or fragments. (A) represents the successful co-transformation of all the prey and bait vectors under double selection (-Trp, -Leu). (B) shows the result of the triple selection (-Trp, -Leu, -His), indicating protein-protein interaction. Since SARS2-Nsp1, Nsp3 (a.a.1–412), Nsp10, Nsp13 (a.a.260–601), and Nsp14 expressed in pGBKT7g bait vector unspecifically bound to the empty prey vector, the experiment of those genes was performed in an exchanged way. Supplementary Figure S2C displays the growth of yeast on a double selection plate in the OSBP yeast 2-hybrid screening assay, parallel to . [file Image_2.jpg]

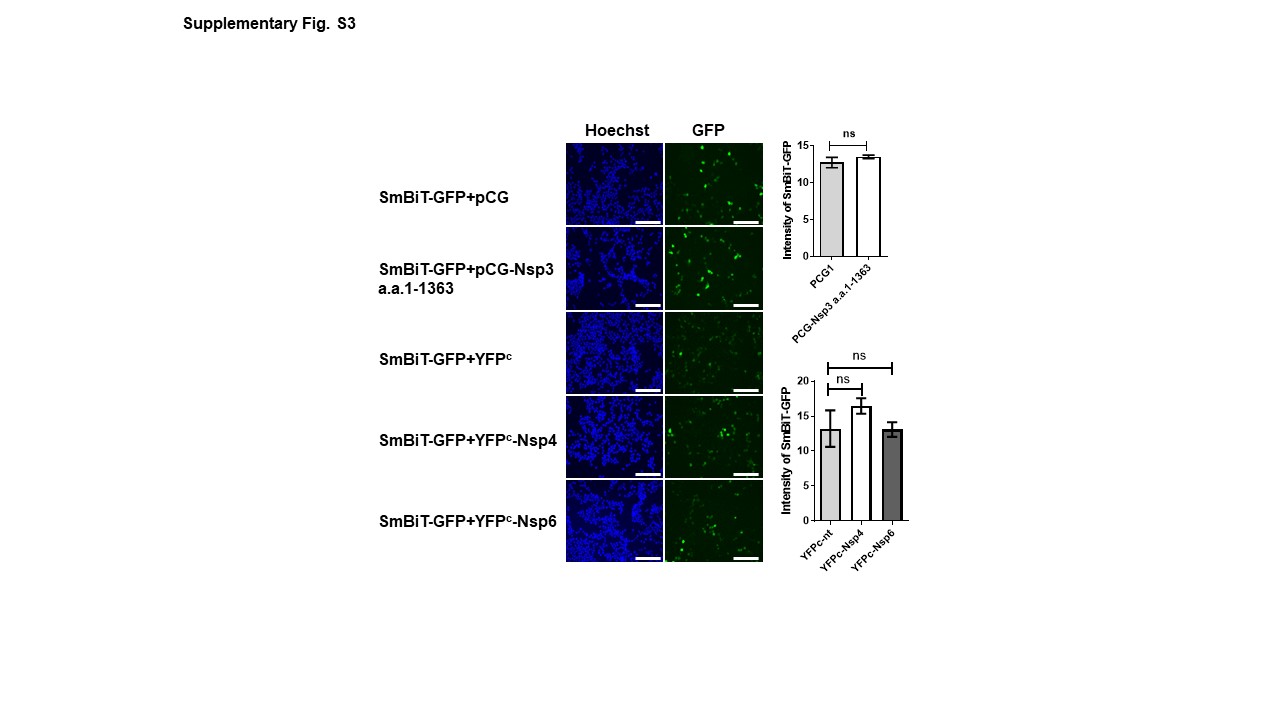

Supplement: Supplementary Figure 3 — SARS-CoV-2 Nsp3 a.a.1–1363, Nsp4, and Nsp6 do not downregulate the HSV TK promoter carried by split-NanoBiT vectors. The indicated plasmids were co-transfected into HEK293 cells in a 24-well plate using Lipofectamine 3000. After 24 hours, the cells were fixed with 4% PFA and stained with Hoechst 33342 (Invitrogen). The intensity of GFP was quantified using ImageJ software. The graphs present mean values and standard deviations calculated from four randomly selected pictures of each sample. Scale bar represents 150 µm. [file Image_3.jpg]

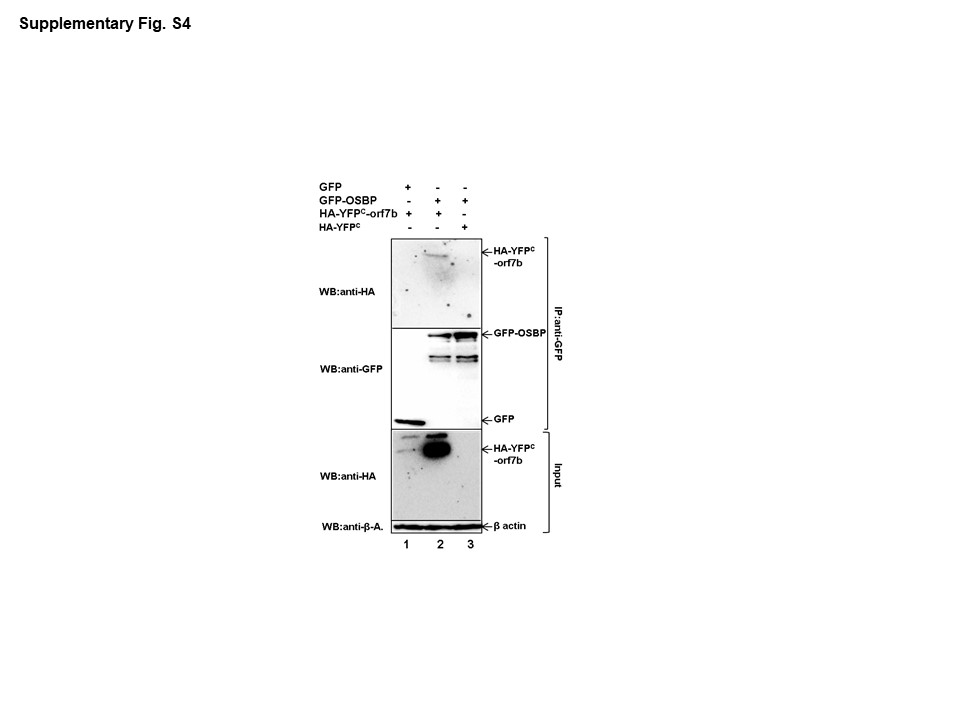

Supplement: Supplementary Figure 4 — OSBP enhances the protein level of SARS-CoV-2 orf7b. Plasmids expressing the indicated constructs pDEST-HA-YFPC-orf7b, pDEST-GFP-OSBP, and their respective controls were transfected into HEK293 cells in a 6-well plate format. After 24 hours, cells were harvested for a GFP-trap-based pulldown assay. [file Image_4.jpeg]

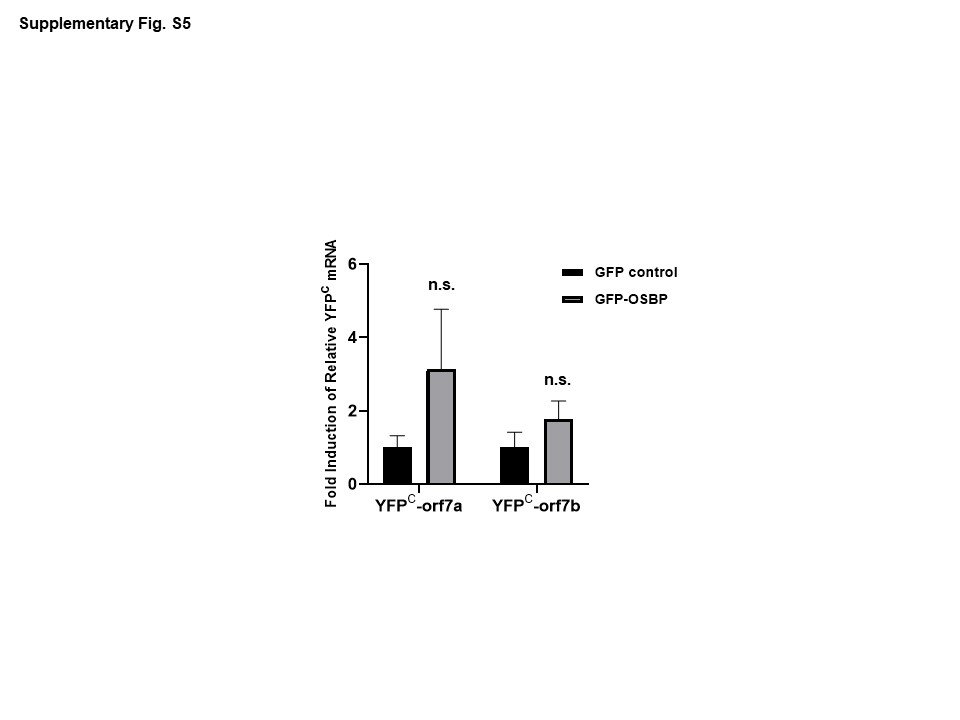

Supplement: Supplementary Figure 5 — OSBP does not regulate SARS-CoV-2 orf7a and orf7b at the mRNA level. HEK293 cells were transfected with pDEST-HA-YFPC-orf7a/-orf7b and either pDEST-GFP control or pDEST-GFP-OSBP plasmid in a 24-well plate. After 24 hours, cellular RNA was isolated for cDNA synthesis and SYBR Green qPCR. The relative YFPC mRNA was calculated as the ratio of YFPC mRNA to its corresponding β-actin mRNA in each sample. Statistical analysis based on biological triplicates showed no significant difference between the GFP control group and the GFP-OSBP group for both relative YFPC-orf7a mRNA and YFPC-orf7b mRNA. The abbreviation ‘n.s.’ was used to indicate that the difference was not significant. [file Image_5.jpeg]

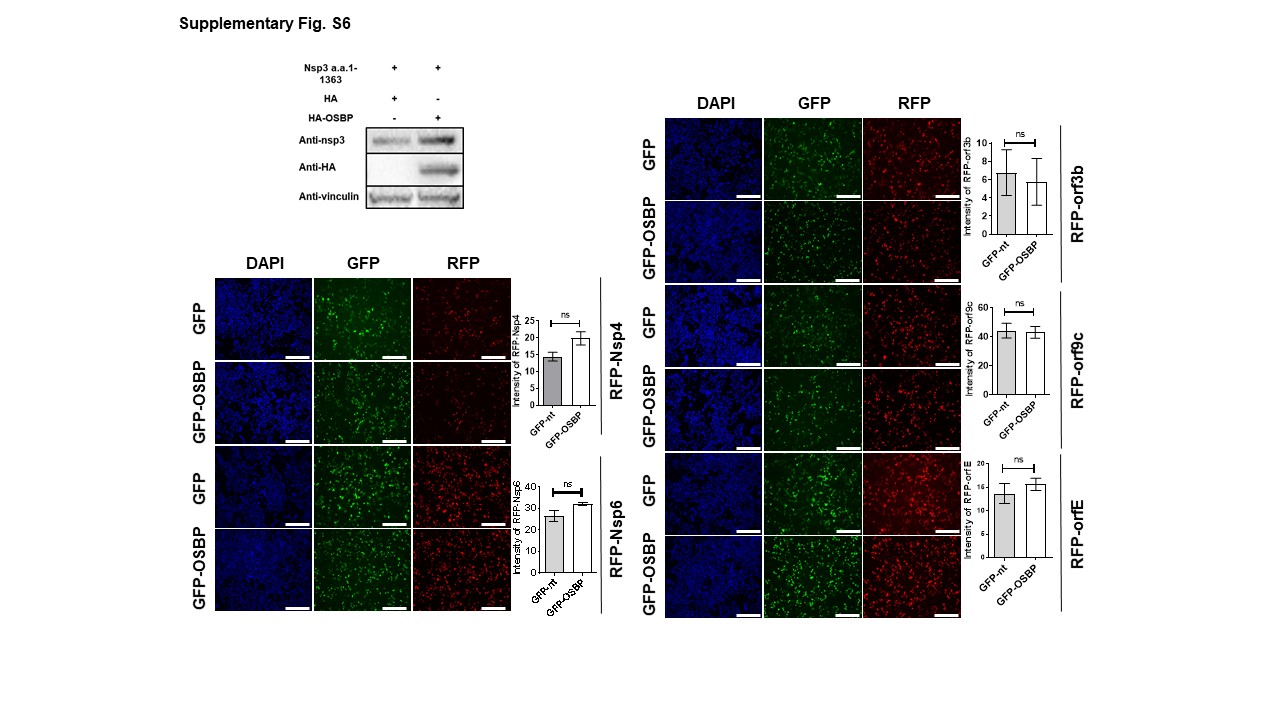

Supplement: Supplementary Figure 6 — OSBP does not affect the protein levels of SARS-CoV-2 Nsp3 a.a.1–1363, Nsp4, Nsp6, orf3b, orf9c, and orfE. Plasmids were constructed as pCG-SARS2-Nsp3 a.a.1–1363, pDest-HA or pDest-HA-OSBP for the Western blot. HEK293 cells were co-transfected with the indicated plasmids in a 12 well-plate using Lipofectamine 3000 and incubated overnight. The protein samples were analyzed with western blot using anti-HA, anti-Nsp3, or anti-vinculin antibodies. Fluorescence microscopy was performed by co-transfecting the indicated plasmids into HEK293 cells in a 24-well plate using Lipofectamine 3000. After 24 hours, cells were fixed and stained with DAPI. Pictures were taken using an EVOS M7000 fluorescence microscope (Thermofisher, 20x objective) and RFP intensity was analyzed using Celleste software on three randomly selected images from each treatment. Scale bar represents 150 µm. [file Image_6.jpg]

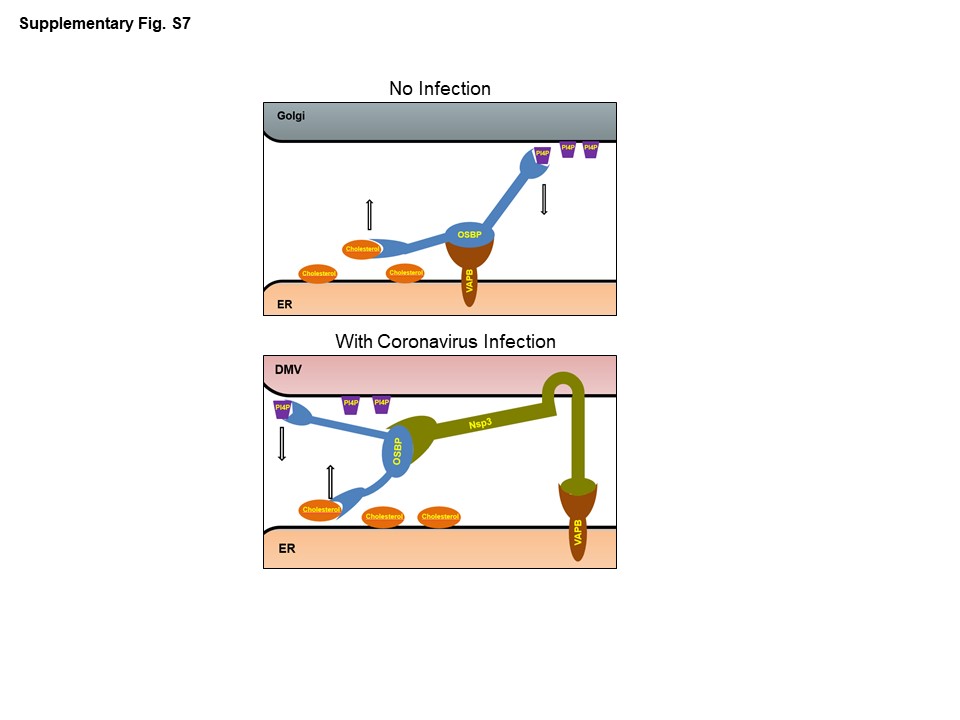

Supplement: Supplementary Figure 7 — Illustration of the process of OSBP-mediated lipid transfer with and without coronavirus infection. In the absence of viral infection, OSBP is anchored by VAPB located in the ER, which regulates the exchange of cholesterol/PI4P between the ER and Golgi apparatus (Mesmin et al., 2013). However, in the presence of coronavirus infection, the interaction between VAPB and OSBP is disrupted by coronaviral Nsp3 as demonstrated in . Nsp3, which is embedded in the DMV membrane (Wolff et al., 2020), interacts with VAPB () to form intimate membrane contact sites between DMVs and ER. Furthermore, Nsp3 interacts with OSBP () to recruit it next to the endoplasmic reticulum (ER) and facilitate lipid transfer from the ER to double-membrane vesicles (DMVs). [file Image_7.jpeg]
