# Supplementary material for: Oxysterole-binding protein targeted by SARS-CoV-2 viral proteins regulates coronavirus replication
Source: Front Cell Infect Microbiol. 2024 Jul 25;14:1383917. doi: 10.3389/fcimb.2024.1383917 (PMC11306179; doi:10.3389/fcimb.2024.1383917)
Supplement: Supplementary Table 1 — Primer list. [file Table_1.docx]

**Table S1. Primer List**

| primer name | primer sequence |
| --- | --- |
| OSBP att for | 5'-GTG GAC AAG TTT GTA CAA AAA AGC AGG CTC CGC CAT GGC GGC GAC GGA GCT GAG AGG AGT GG-3' |
| OSBP att rev | 5'-GGG GAC CAC TTT GTA CAA GAA AGC TGG GTC TCM GAA AAT GTC CGG GCA TGA GCT CCA GTC CTG-3' |
| OSBP gRNA1 for | 5’-CAC CGA TGG CGG CGA CGG AGC TGA G-3’ |
| OSBP gRNA1 rev | 5’-AAA CCT CAG CTC CGT CGC CGC CAT C-3’ |
| OSBP gRNA2 for | 5’-CAC CGG GCG ACG GAG CTG AGA GGA G-3‘ |
| OSBP gRNA2 rev | 5’-AAA CCT CCT CTC AGC TCC GTC GCC C-3’ |
| VAP-B EcoRI for | 5'-GTG GAG CTC AGG GGA ATT CAA TGG CGA AGG TGG AGC-3' |
| VAPB XbaI rev | 5'-GCC GCC CCG ACT CTA GAC TAC AAG GCA ATC TTC CC-3' |
| rfB XhoI for | 5'- GCG CGC TCG AGA TCA ACA AGT TTG TAC-3' |
| rfB XbaI rev | 5'- GCT GCT CTA GAA CCA CTT TGT ACA AGA AAG C-3' |
| HCoV-OC43 qPCR for | 5'-CGC CGC CTT ATT AAA GAT GTT G-3' |
| HCoV-OC43 qPCR rev | 5'-GGC ATA GCA CGA TCA CAC TTA GG-3' |
| HCoV-OC43 qPCR probe | 5'-FAM-AAT CCT GTA CTT ATG GGT TGG GAT T-BHQ1-3' |
| Sybr Green HA-YFP^C^ for | 5'-GAG AGA CCG GTA CTA GTA TGT ACC CAT ACG ATG TTC C-3' |
| Sybr Green HA-YFP^C^ rev | 5'-ATA AGG GCC CTT AAC TCG AGA ACT TGT ACA GCT CGT CCA TG-3' |
| Sybr Green β-actin for | 5'-ATA TAG GCC GTC TTC CCC TCC ATC G-3' |
| Sybr Green β-actin rev | 5'-ATG GAG TCC ATC ACG ATG CCA GTG-3' |
| GFP att for | 5'-TTC TAC AAG TTT GTA CAA AAA AGC AGG CTC CGC CAT GAG CGG GGG CGA GGA GCT GTT CGC C-3' |
| GFP att rev | 5'-ACA TAC CAC TTT GTA CAA GAA AGC TGG GTC TCM CTC GTC CAT GCC GTG GGT GTG GCA GCA G-3' |
| SARS-CoV-2 nsp1 att for | 5'-GGG GAC AAG TTT GTA CAA AAA AGC AGG CTC CGC CAT GGA GAG CCT TGT CCC TGG-3' |
| SARS-CoV-2 nsp1 att rev | 5'-GGG GAC CAC TTT GTA CAA GAA AGC TGG GTC TCM CCC TCC GTT AAG CTC ACG CAT G-3' |
| SARS-CoV-2 nsp2 att for | 5'-GGG GAC AAG TTT GTA CAA AAA AGC AGG CTC CGC CAT GGC ATA CAC TCG CTA TGT CG-3' |
| SARS-CoV-2 nsp2 att rev | 5'-GGG GAC CAC TTT GTA CAA GAA AGC TGG GTC TCM ACC GCC TTT GAG TGT GAA GG-3' |
| SARS-CoV-2 nsp3 (aa1-412) att for | 5'-GGG GAC AAG TTT GTA CAA AAA AGC AGG CTC CGC CAT GGC ACC AAC AAA GGT TAC TTT TGG TGA TG-3' |
| SARS-CoV-2 nsp3 (aa1-412) att rev | 5'-GGG GAC CAC TTT GTA CAA GAA AGC TGG GTC TCM CTT ATC ATC TTG TTT TCT CTG TTC AAC TG-3' |
| SARS-CoV-2 nsp3-PLP att for | 5'-GGG GAC AAG TTT GTA CAA AAA AGC AGG CTC CGC CAT GGA AGT GAG GAC TAT TAA GGT GTT TAC AAC-3' |
| SARS-CoV-2 nsp3-PLP att rev | 5'-GGG GAC CAC TTT GTA CAA GAA AGC TGG GTC TCM ATA AGT AAC TGG TTT TAT GGT TGT TGT GTA ACT G-3' |
| SARS-CoV-2 nsp3-SUD att for | 5'-GGG GAC AAG TTT GTA CAA AAA AGC AGG CTC CGC CAT GAA AAT CAA AGC TTG TGT TGA AGA AGT TAC-3' |
| SARS-CoV-2 nsp3-SUD att rev | 5'-GGG GAC CAC TTT GTA CAA GAA AGC TGG GTC TCM AGA AAG AAG TGT CTT AAG ATT GTC AAA GG-3' |
| SARS-CoV-2 nsp3 (aa1065-1414) att for | 5'-GGG GAC AAG TTT GTA CAA AAA AGC AGG CTC CGC CAT GAA ATT GGA TGG TGT TGT TTG TAC AGA AAT TG-3' |
| SARS-CoV-2 nsp3 (aa1065-1414) att rev | 5'-GGG GAC CAC TTT GTA CAA GAA AGC TGG GTC TCM CCA AAT TAT AAT ATT TAT CAG TTT AGA AAA ATT AGG-3' |
| SARS-CoV-2 nsp3 (aa1547-1945) att for | 5'-GGG GAC AAG TTT GTA CAA AAA AGC AGG CTC CGC CAT GTG GTT AAT AAT TAA TCT TGT ACA AAT G-3' |
| SARS-CoV-2 nsp3 (aa1547-1945) att rev | 5'-GGG GAC CAC TTT GTA CAA GAA AGC TGG GTC TCM ACC ACC CTT AAG TGC TAT CTT TGT TGT TAC-3' |
| SARS-CoV-2 nsp4 att for | 5'-GGG GAC AAG TTT GTA CAA AAA AGC AGG CTC CGC CAT GAA AAT TGT TAA TAA TTG GTT GAA GCA G-3' |
| SARS-CoV-2 nsp4 att rev | 5'-GGG GAC CAC TTT GTA CAA GAA AGC TGG GTCT CMC TGC AAA ACA GCT GAG GTG ATA G-3' |
| SARS-CoV-2 nsp5 att for | 5'-GGG GAC AAG TTT GTA CAA AAA AGC AGG CTC CGC CAT GAG TGG TTT TAG AAA AAT GGC ATT CC-3' |
| SARS-CoV-2 nsp5 att rev | 5'-GGG GAC CAC TTT GTA CAA GAA AGC TGG GTC TCM TTG GAA AGT AAC ACC TGA GCA TTG-3' |
| SARS-CoV-2 nsp6 att for | 5'-GGG GAC AAG TTT GTA CAA AAA AGC AGG CTC CGC CAT GAG TGC AGT GAA AAG AAC AAT CAA GG-3' |
| SARS-CoV-2 nsp6 att rev | 5'-GGG GAC CAC TTT GTA CAA GAA AGC TGG GTC TCM CTG TAC AGT GGC TAC TTT GAT AC-3' |
| SARS-CoV-2 nsp7 att for | 5'-GGG GAC AAG TTT GTA CAA AAA AGC AGG CTC CGC CAT GTC TAA AAT GTC AGA TGT AAA GTG CAC-3' |
| SARS-CoV-2 nsp7 att rev | 5'-GGG GAC CAC TTT GTA CAA GAA AGC TGG GTC TCM TTG TAA GGT TGC CCT GTT GTC-3' |
| SARS-CoV-2 nsp8 att for | 5'-GGG GAC AAG TTT GTA CAA AAA AGC AGG CTC CGC CAT GGC TAT AGC CTC AGA GTT TAG TTC C-3' |
| SARS-CoV-2 nsp8 att rev | 5'-GGG GAC CAC TTT GTA CAA GAA AGC TGG GTC TCM CTG TAA TTT GAC AGC AGA ATT GGC-3' |
| SARS-CoV-2 nsp9 att for | 5'-GGG GAC AAG TTT GTA CAA AAA AGC AGG CTC CGC CAT GAA TAA TGA GCT TAG TCC TGT TGC AC-3' |
| SARS-CoV-2 nsp9 att rev | 5'-GGG GAC CAC TTT GTA CAA GAA AGC TGG GTC TCM TTG TAG ACG TAC TGT GGC AGC-3' |
| SARS-CoV-2 nsp10 att for | 5'-GGG GAC AAG TTT GTA CAA AAA AGC AGG CTC CGC CAT GGC TGG TAA TGC AAC AGA AGT GC-3' |
| SARS-CoV-2 nsp10 att rev | 5'-GGG GAC CAC TTT GTA CAA GAA AGC TGG GTC TCM CTG AAG CAT GGG TTC GCG G-3' |
| SARS-CoV-2 nsp12-niran att for | 5'-GGG GAC AAG TTT GTA CAA AAA AGC AGG CTC CGC CAT GTC AGC TGA TGC ACA ATC GTT TTT AAA CCG GGT TTG CGG TGT AAG TG-3' |
| SARS-CoV-2 nsp12-niran att rev | 5'-GGG GAC CAC TTT GTA CAA GAA AGC TGG GTC TCM CCT GGT CAA GGT TAA TAT AGG CAT TAA C-3' |
| SARS-CoV-2 nsp12-interface att for | 5'-GGG GAC AAG TTT GTA CAA AAA AGC AGG CTC CGC CAT GGC TTT AAC TGC AGA GTC ACA TG-3' |
| SARS-CoV-2 nsp12-interface att rev | 5'-GGG GAC CAC TTT GTA CAA GAA AGC TGG GTC TCM AAA GCA CGT AGT GCG TTT ATC TAG-3' |
| SARS-CoV-2 nsp12-Rdrp att for | 5'-GGG GAC AAG TTT GTA CAA AAA AGC AGG CTC CGC CAT GTC AGT AGC TGC ACT TAC TAA CAA TG-3' |
| SARS-CoV-2 nsp12-Rdrp att rev | 5'-GGG GAC CAC TTT GTA CAA GAA AGC TGG GTC TCM CTG TAA GAC TGT ATG CGG TGT GTA CAT AGC C-3' |
| SARS-CoV-2 nsp13 (aa1-259) att for | 5'-GGG GAC AAG TTT GTA CAA AAA AGC AGG CTC CGC CAT GGC TGT TGG GGC TTG TGT TCT TTG-3' |
| SARS-CoV-2 nsp13 (aa1-259) att rev | 5'-GGG GAC CAC TTT GTA CAA GAA AGC TGG GTC TCM TGA GAT ATT GAG TGT TGG GTA TAA GCC AG-3' |
| SARS-CoV-2 nsp13 (aa260-601) att for | 5'-GGG GAC AAG TTT GTA CAA AAA AGC AGG CTC CGC CAT GGA TGA GTT TTC TAG CAA TGT TGC AAA TTA TC-3' |
| SARS-CoV-2 nsp13 (aa260-601) att rev | 5'-GGG GAC CAC TTT GTA CAA GAA AGC TGG GTC TCM TTG TAA AGT TGC CAC ATT CCT ACG TG-3' |
| SARS-CoV-2 nsp14 att for | 5'-GGG GAC AAG TTT GTA CAA AAA AGC AGG CTC CGC CAT GGC TGA AAA TGT AAC AGG ACT CTT TAA AG-3' |
| SARS-CoV-2 nsp14 att rev | 5'-GGG GAC CAC TTT GTA CAA GAA AGC TGG GTC TCM CTG AAG TCT TGT AAA AGT GTT CCA GAG-3' |
| SARS-CoV-2 nsp15 att for | 5'-GGG GAC AAG TTT GTA CAA AAA AGC AGG CTC CGC CAT GAG TTT AGA AAA TGT GGC TTT TAA TGT TG-3' |
| SARS-CoV-2 nsp15 att rev | 5'-GGG GAC CAC TTT GTA CAA GAA AGC TGG GTC TCM TTG TAA TTT TGG GTA AAA TGT TTC TAC ATG GC-3' |
| SARS-CoV-2 nsp16 att for | 5'-GGG GAC AAG TTT GTA CAA AAA AGC AGG CTC CGC CAT GTC TAG TCA AGC GTG GCA ACC G-3' |
| SARS-CoV-2 nsp16 att rev | 5'-GGG GAC CAC TTT GTA CAA GAA AGC TGG GTC TCM GTT GTT AAC AAG AAC ATC ACT AGA AAT AAC AAC-3' |
| SARS-CoV-2 orf3a att for | 5'-GGG GAC AAG TTT GTA CAA AAA AGC AGG CTC CGC CAT GGA TTT GTT TAT GAG AAT CTT CAC AAT TGG-3' |
| SARS-CoV-2 orf3a att rev | 5'-GGG GAC CAC TTT GTA CAA GAA AGC TGG GTC TCM CAA AGG CAC GCT AGT AGT CGT C-3' |
| SARS-CoV-2 orf3b att for | 5'-GGG GAC AAG TTT GTA CAA AAA AGC AGG CTC CGC CAT GGC TTA TTG TTG GCG TTG C-3' |
| SARS-CoV-2 orf3b att rev | 5'-GGG GAC CAC TTT GTA CAA GAA AGC TGG GTC TCM AGG CCA GCA GCA ACG AGC-3' |
| SARS-CoV-2 orf6 att for | 5'-GGG GAC AAG TTT GTA CAA AAA AGC AGG CTC CGC CAT GTT TCA TCT CGT TGA CTT TCA GG-3' |
| SARS-CoV-2 orf6 att rev | 5'-GGG GAC CAC TTT GTA CAA GAA AGC TGG GTC TCM ATC AAT CTC CAT TGG TTG CTC-3' |
| SARS-CoV-2 orf7a att for | 5'-GGG GAC AAG TTT GTA CAA AAA AGC AGG CTC CGC CAT GAT GAA AAT TAT TCT TTT CTT GGC ACT GAT AAC AC-3' |
| SARS-CoV-2 orf7a att rev | 5'-GGG GAC CAC TTT GTA CAA GAA AGC TGG GTC TCM TTC TGT CTT TCT TTT GAG TGT GAA GC-3' |
| SARS-CoV-2 orf7b att rev | 5'-GGG GAC AAG TTT GTA CAA AAA AGC AGG CTC CGC CAT GAT TGA ACT TTC ATT AAT TGA CTT CTA TTT GTG C-3' |
| SARS-CoV-2 orf7b att rev | 5'-GGG GAC CAC TTT GTA CAA GAA AGC TGG GTC TCM GGC GTG ACA AGT TTC ATT ATG ATC TTG C-3' |
| SARS-CoV-2 orf8 att for | 5'-GGG GAC AAG TTT GTA CAA AAA AGC AGG CTC CGC CAT GAA ATT TCT TGT TTT CTT AGG AAT CAT CAC AAC TG-3' |
| SARS-CoV-2 orf8 att rev | 5'-GGG GAC CAC TTT GTA CAA GAA AGC TGG GTC TCM GAT GAA ATC TAA AAC AAC ACG AAC GTC ATG-3' |
| SARS-CoV-2 orf9c att for | 5'-GGG GAC AAG TTT GTA CAA AAA AGC AGG CTC CGC CAT GCT GCA ATC GTG CTA CAA CTT C-3' |
| SARS-CoV-2 orf9c att rev | 5'-GGG GAC CAC TTT GTA CAA GAA AGC TGG GTC TCM ATC TGT CAA GCA GCA GCA AAG C-3' |
| SARS-CoV-2 orf10 att for | 5'-GGG GAC AAG TTT GTA CAA AAA AGC AGG CTC CGC CAT GGG CTA TAT AAA CGT TTT CGC TTT TCC-3' |
| SARS-CoV-2 orf10 att rev | 5'-GGG GAC CAC TTT GTA CAA GAA AGC TGG GTC TCM TGT GAG ATT AAA GTT AAC TAC ATC TAC TTG TGC-3' |
| SARS-CoV-2 orfE att for | 5'-GGG GAC AAG TTT GTA CAA AAA AGC AGG CTC CGC CAT GTA CTC ATT CGT TTC GGA AGA GAC-3' |
| SARS-CoV-2 orfE att rev | 5'-GGG GAC CAC TTT GTA CAA GAA AGC TGG GTC TCM GAC CAG AAG ATC AGG AAC TCT AGA AG-3' |
| SARS-CoV-2 orfM att for | 5'-GGG GAC AAG TTT GTA CAA AAA AGC AGG CTC CGC CAT GGC AGA TTC CAA CGG TAC TAT TAC-3' |
| SARS-CoV-2 orfM att rev | 5'-GGG GAC CAC TTT GTA CAA GAA AGC TGG GTC TCM CTG TAC AAG CAA AGC AAT ATT GTC AC-3' |
| SARS-CoV-2 orfN att for | 5'-GGG GAC AAG TTT GTA CAA AAA AGC AGG CTC CGC CAT GTC TGA TAA TGG ACC CC-3' |
| SARS-CoV-2 orfN att rev | 5'-GGG GAC CAC TTT GTA CAA GAA AGC TGG GTC TCM GGC CTG AGT TGA GTC AGC AC-3' |
| SARS-CoV-2 S1 (1-329) att for | 5'-GGG GAC AAG TTT GTA CAA AAA AGC AGG CTC CGC CAT GTT TGT TTT TCT TGT TTT ATT GCC ACT AG-3' |
| SARS-CoV-2 S1 (1-329) att rev | 5'-GGG GAC CAC TTT GTA CAA GAA AGC TGG GTC TCM AAA TCT AAC AAT AGA TTC TGT TGG TTG-3' |
| SARS-CoV-2 S-RBD att for | 5'-GGG GAC AAG TTT GTA CAA AAA AGC AGG CTC CGC CAT GTC TAT TGT TAG ATT TCC TAA TAT TAC AAA CTT GTG CC-3' |
| SARS-CoV-2 S-RBD att rev | 5'-GGG GAC CAC TTT GTA CAA GAA AGC TGG GTC TCM TGT AAT GTC AAG AAT CTC AAG TGT CTG TG-3' |
| SARS-CoV-2 S2 att for | 5'-GGG GAC AAG TTT GTA CAA AAA AGC AGG CTC CGC CAT GTG TGA CAT ACC CAT TGG TGC AGG TAT ATG C-3' |
| SARS-CoV-2 S2 att rev | 5'-GGG GAC CAC TTT GTA CAA GAA AGC TGG GTC TCM TGT GTA ATG TAA TTT GAC TCC TTT GAG CAC-3' |
| SARS-CoV-2 S2 aa1205-1254 deletion for | 5'-CTT TCC AAG TTC TTG GAG ATC-3' |
| SARS-CoV-2 S2 aa1205-1254 deletion rev | 5'-AAA TTT GAT GAA GAC GAC TCT G-3' |
